# Capturing tumor complexity *in vitro*: Comparative analysis of 2D and 3D tumor models for drug discovery

**DOI:** 10.1038/srep28951

**Published:** 2016-07-01

**Authors:** Kristin Stock, Marta F. Estrada, Suzana Vidic, Kjersti Gjerde, Albin Rudisch, Vítor E. Santo, Michaël Barbier, Sami Blom, Sharath C. Arundkar, Irwin Selvam, Annika Osswald, Yan Stein, Sylvia Gruenewald, Catarina Brito, Wytske van Weerden, Varda Rotter, Erwin Boghaert, Moshe Oren, Wolfgang Sommergruber, Yolanda Chong, Ronald de Hoogt, Ralph Graeser

**Affiliations:** 1 Bayer Pharma AG, Muellerstr. 178, 13353 Berlin, Germany; 2 iBET, Instituto de Biologia Experimental e Tecnológica, Apartado 12, 2780-901 Oeiras, Portugal; 3 Instituto de Tecnologia Química e Biológica António Xavier, Universidade Nova de Lisboa, Av. da República, 2780-157 Oeiras, Portugal; 4 Janssen Pharmaceutica nv, Turnhoutseweg 30, 2340 Beerse, Belgium; 5 ERASMUS MC, Wytemaweg 80, 3015 CN Rotterdam, The Netherlands; 6 Faculty of Mathematics, Natural Sciences and Information Technologies, University of Primorska, Koper, Slovenia; 7 Boehringer Ingelheim RCV, GmbH & Co. KG, Doktor-Boehringer-Gasse 5-11, 1120 Wien, Austria; 8 AbbVie, 1 North Waukegan Road, North Chicago, IL 60064-6098, USA; 9 Institute for Molecular Medicine Finland FIMM, Tukholmankatu 8, 00014 University of Helsinki, Finland; 10 The Weizmann Institute, 234 Herzl St., Rehovot 7610001, Israel; 11 Institute of Medical Genetics, Medical University of Vienna, Waehringerstrasse 10, A-1090 Vienna, Austria; 12 Boehringer Ingelheim Pharma GmbH & Co. KG, Birkendorfer Str. 65, 88400 Biberach an der Riß, Germany

## Abstract

Two-dimensional (2D) cell cultures growing on plastic do not recapitulate the three dimensional (3D) architecture and complexity of human tumors. More representative models are required for drug discovery and validation. Here, 2D culture and 3D mono- and stromal co-culture models of increasing complexity have been established and cross-comparisons made using three standard cell carcinoma lines: MCF7, LNCaP, NCI-H1437. Fluorescence-based growth curves, 3D image analysis, immunohistochemistry and treatment responses showed that end points differed according to cell type, stromal co-culture and culture format. The adaptable methodologies described here should guide the choice of appropriate simple and complex *in vitro* models.

Despite the increase in the survival rates for many cancers over the past four decades, the discovery of novel effective drugs has decreased^1^. Overall, the success rate of novel oncology drugs transitioning in the clinic from phase 2 to phase 3 is low^2^. A lack of efficacy was suggested as a main reason for failure^3^. Since novel drugs are propelled to clinical trial based on evidence of efficacy in preclinical models, clearly these models are questionable.

Despite the wealth of data generated, and strong recommendations to upgrade cell culture from 2D to 3D models^4^, few of these more complex model systems have been incorporated into the drug discovery funnel. Reproducibility, cost, time to set up, and limited throughput are some of the issues precluding their routine use. Importantly, a lack of detailed characterization and cross-comparison of complex models to show added value relative to simple 2D models is absent from many published studies. Thus there is still a need for a better understanding of these complex models in order to define their utility and limitations so as to then place them in a more comprehensive drug discovery cascade.

The PREDECT consortium (www.predect.eu) has assumed the task to compare and better characterize *in vitro* models for oncology research, particularly models that attempt to capture the complexity and heterogeneity of human cancers^5^. Models were set up for three pathologies, breast, prostate, and lung carcinomas. For breast and prostate cancer models, MCF7 and LNCaP cell lines were chosen due to their responsiveness to anti-hormone treatment as a standard of care (SOC). The lung adenocarcinoma cell line H1437 is sensitive to the pan-PI3 kinase/mTOR inhibitor GSK1059615 as the positive control targeted agent selected for the lung pathology^6^. Normal (NF) and cancer-associated fibroblasts (CAF) served to represent the local stroma for prostate and lung cancer models^7^,^8^. For breast cancer models, no stable breast cancer derived fibroblast cell lines could be obtained, and human non-immortalized dermal fibroblasts (HDF) served as the stromal cell compartment. Albeit not breast-derived, HDFs are specialized in producing collagen^9^, which is a predominant ECM component in breast cancer^10^. In previous publications, we have demonstrated that these fibroblasts contribute to a pro-inflammatory and pro-angiogenic environment^11^,^12^. Indeed, fibroblasts may be defined by their functionality rather than by their origin^13^, and HDFs may functionally be re-programmed by tumor cells to become CAFs^14^. Thus given their ready availability, greater robustness, and suggested functional adequacy, we decided to use HDFs as a surrogate for breast-derived CAFs.

Starting from simple 2D monocultures, the complexity was increased stepwise to include stromal cells in 2D co-cultures, and then growth of the cultures in 3D. 3D cultures were set up either as free-floating spheroids (“floaters”), microencapsulated into inert hydrogels (alginate) and grown in bioreactors (“alginate-BR”), or embedded in ECM, all in the presence or absence of stromal cells. ECM embedded cultures were established in, (1) Matrigel, a basal membrane extract that induces polarization of normal epithelial cells^15^, and would thus reflect a localized tumor environment, (2) collagen I as an interstitial stroma matrix component, providing an invasive growth environment^16^, and, (3) a 1:1 mix of both. The alginate hydrogel capsules used in the alginate-BR models show some similarity to the glycosaminoglycan structure present in the stromal compartment, such as the ability to form gels at very low concentrations, attract a cloud of cations, such as Na^+^ or Ca^2+^ and incorporate high amounts of water into the matrix^17^. In addition, the inert structure keeps tumor spheroids and stromal cells in close proximity, and may be model-tailored by ECM depositions from the embedded cells. In contrast to the other models, stirred-tank bioreactors allow for precise control of physicochemical parameters such as pH, O_2_ and perfusion rates.

Cell growth was monitored in all models by fluorescence measurements. Also, response to standard of care (SOC) hormonal treatment (breast/prostate) or targeted treatment (lung) and chemotherapy (all) was measured and compared between all models. When stationary growth phase was reached, cultures were analyzed in more depth by fluorescence imaging of *in situ* fixed cultures, as well as immunohistochemistry (IHC) on paraffin embedded samples processed into tissue micro-arrays (TMAs). The decision to choose imaging methods that leave the cellular organization of the models intact rather than disruptive technologies like genomic or transcriptional profiling, which may provide higher throughput, was taken considering the increasingly recognized role of tumor cell heterogeneity in drug response and resistance. The robust protocols established by this collaborative effort in combination with the cross-comparisons performed to characterize the models provide a toolbox that should help to better incorporate complex models in the drug discovery process.

## Materials and Methods

### Experimental procedures

#### Cell lines, passaging, and viral transductions

Cells were passaged according to guidelines from ATCC or the cell provider (see Table 1, also for the fluorescence tags).

MCF-7 cells, transduced with the lentiviral vectors PGK-GFP and pCDH-CMV-MCS-EF1-Puro, were kindly provided by Professor Cathrin Brisken (EPFL, Switzerland), within the scope of the PREDECT consortium. Human dermal fibroblasts (HDF) were transfected with tag-RFP, as described in ref. 12.

Dual function lentiviral vectors pLEX_TRC211/L2N-TurboRFP-c and pLEX_TRC203/CMV-Ren-puro-eGFP were generated carrying a huEF1α promoter to drive either a tRFP, or a Renilla luciferase (RLuc)-eGFP fusion reporter. The former construct also harbors a firefly luciferase (FLuc) cDNA fused to the neomycin resistance ORF under the control of PGK promoter. Lentiviral particles were produced by co-transfecting three plasmids (packaging plasmid psPAX2 (300 ng/well), VSV-G expressing plasmid pMD2.G (30 ng/well) and the vector of interest (270 ng/well)) into HEK-293T cells in 6-well tissue culture plates, using lipopolyplex TransIT-LT1 Transfection Reagent (Mirus Bio, MIR2305). After 24 hours, complete culture medium (DMEM medium without phenol red, supplemented with 10% FBS, 0.04% gentamycin and 1% glutamine) was replaced with medium containing 10% BSA. Five days post transfection, the culture supernatant was harvested, aliquoted and frozen. Prostate cancer and stromal cells were seeded in 6-well tissue culture plates containing 2 ml culture medium/well. One day after, cells were infected with 1 ml viral supernatant in the presence of 8 μg/ml polybrene (Sigma), centrifuged at 2200 rpm for 90 min and incubated at 37 °C, 5% CO_2_ for 24 h, after which the medium was refreshed. Transduced prostate cancer and stromal cells were selected and sorted for uniform fluorescence by flow cytometry (BD FACSAria).

NCI-H1437 cells were transduced with a lentiviral construct expressing tomato-RFP (Capital Biosciences, Inc.). Lung derived Normal (NF) and Cancer Associated Fibroblasts (CAF) from patient number 4731 were isolated as described previously^18^. The local ethics committee ‘Ethik-Kommission der Medizinischen Fakultät am Universitätsklinikum Tübingen’ approved the study (project number 396/2005V and 159/2011BO2) and a written informed consent was obtained from the patient. NF and CAF were immortalized using a virus co-expressing hTERT and GFP (Lenti-hTERT-eGFP; Cat No- LG508, BioGenova), as described in ref. 8.

#### Culture set-up

##### 2D culture

Cells were seeded in black 96-well clear-bottom microplates (Greiner Bio-One #655-088). For cell numbers, medium, and FCS concentration see Table 2. The plates were incubated in a humidified atmosphere with 5% CO_2_ at 37 °C.

##### Floater culture

Cells were seeded in 100–200 μl into low attachment U-bottom 96-well plates (Corning #4520), or in 40 μl of medium into 1.5% (w/v) agarose-coated 384-well plates (Greiner #781090), or 50 μl of medium into low attachment U-bottom 384-well plates (Corning #3830)^19^. See Table 2 for cell numbers, medium, and FCS concentration. The plates were incubated in a humidified atmosphere with 5% CO_2_ at 37 °C.

##### 3D alginate-embedded stirred-tank Bioreactor cultures (alginate-BR)

Tumor cells were inoculated as single cell suspensions at a density of 2 × 10^5^ cell/ml into 125 ml stirred tank bioreactors (spinner vessels with straight blade paddle impeller, Corning® Life Sciences, BR). Cell aggregation and spheroid size were controlled through manipulation of the stirring rate^20^. Once compact and spherical shaped spheroids were attained (e.g. 24 h for MCF7 and 48 h for H1437 cells), the tumor spheroids were microencapsulated in alginate with or without fibroblasts as single cells at a 1:1 cell ratio^11^. The resulting microcapsules, with 500 μm of diameter, were transferred to 125 ml stirred-tank bioreactors and cultured at 80 rpm. Cultures were grown in humidified incubators with 5% CO_2_ and 21% O_2_, with 50% medium exchange every 3–4 days.

##### 3D embedded culture

3D cultures were embedded in Matrigel (Corning 356231, Lot 3198769, growth factor reduced, phenol red, and LDEV-free), Collagen Type I (BD Bioscience 354236, Lot 07484), or a 1:1 mix between the two. Collagen was neutralized according to Artym and Matsumoto^21^. A pH in the range 7.1–7.4 was crucial for cells to grow in collagen. Matrigel was used at a final concentration of 4 mg/ml, collagen at 1.5 mg/ml, and the mix contained 4 mg/ml Matrigel and 1.5 mg/ml collagen. Cells were suspended in the appropriate matrix (for cell numbers, medium, and FCS concentration see Table 2) and seeded in 60 μl/well into black 96-well plates (Greiner, #655090), pre-coated with the respective matrix (30 μl/well). Matrix without cells served as a background control. The matrix was allowed to set for 30 min at 37 °C before adding 90 μl growth medium into each well. Growth medium was carefully removed and replenished every 3 days.

Tumor and stromal cell growth was followed via fluorescence measurements of their respective fluorescent protein markers (see Table 1), using a plate reader (see Table S1 for details). Readings from each well were normalized to the reading at day 1 and averaged for each condition. Every condition was run at least in triplicate.

#### Standard-of-care (SOC) treatment

Drug treatment started when cells entered the exponential growth phase.

In a first experiment, IC_50_ and IC_80_ values were determined for Fulvestrant (ICI 182,780), MDV3100, and GSK1059615 for all culture formats, using fluorescence as a read-out for growth (drug concentration ranges are listed in Table S2).

For alginate-BR cultures, microencapsulated mono and co-cultures were collected from the stirred-tank and plated in 96-well plates, with approximately 10 aggregates per well, under orbital agitation. Treatments lasted 4 days for Docetaxel and GSK1059615 and 10 days for Fulvestrant with medium and drug replenishment every 3–4 days. Cell viability was determined with CellTiter-Glo® 3D Cell Viability Assay (Promega). For total cell lysis, aggregates were incubated with the CellTiter-Glo® reagent for 40 min under strong agitation, pipetted up and down quickly 10 times each well, and placed under strong agitation for another 40 min.

In a second experiment, fixed drug concentrations (IC_80_ for targeted therapy drugs (Fulvestrant (ICI 182,780), MDV3100, and GSK1059615) and IC_50_ for Docetaxel; see Table S2) or an equivalent volume of DMSO as vehicle control were selected for growth curves and sample analysis at endpoint.

Tumor and stromal cell growth was followed via fluorescence measurements as above.

#### Fluorescence *in situ* microscopy and analysis

At the endpoint, samples of all 3D models were fixed *in-situ* and imaged via fluorescence microscopy. Alginate-BR and embedded 3D cultures were additionally incubated with 10 μM Click-iT® 5-ethynyl-2′-deoxyuridine (EdU) Alexa Fluor 647 (Life Technologies #C10356) for 2 h and 5 μg/ml Hoechst 33342 (Invitrogen, H3570) for the last 30 min before fixation.

Images were acquired by bright-field and confocal microscopy, depending on the type of analysis. Microscopes, lenses, and settings are summarized in Table S1.

For visualization purposes, the z-stacks, with inter-slice z-distances of 10 μm for embedded samples (except for lung) and 1 μm for bioreactor samples, are shown in maximum intensity z-projections, with all channels merged as RGB values. Linear brightness and contrast adjustments of the images were performed using the ImageJ open source software^22^.

Analysis of fluorescent cultures was a multi-step procedure, consisting of: (1) image acquisition as 3D image stacks; (2) annotation of culture parameters; (3) image processing & feature acquisition, done by a dedicated (semi-)automated 3D image analysis procedure (described in ref. 23), which includes the segmentation of the tumor cell spheroids, detection of EdU positive cells within the spheroids, and extraction of features pertaining to the size, shape, number of the cancer spheroids; and, (4) data analysis of the features using dedicated R-scripts.

Exceptions to this procedure were necessary for size and shape analysis of alginate-BR and floater cultures, for which 2D images were analyzed using dedicated CellProfiler pipelines. Also, for the quantification of EdU-positive cells in alginate-BR cultures, 2D spheroid masks were drawn manually using the RoiManager plugin in ImageJ.

#### Paraformaldehyde fixation and paraffin-embedding

3D floater, alginate-BR, and collagen or Matrigel/collagen mixed embedded cultures were fixed and paraffin-embedded for TMA analysis. For the floater cultures, 100 spheroids were collected into a falcon tube, washed with PBS and fixed in 5 ml 4% (w/v) paraformaldehyde (PFA, Sigma) for 15 min at room temperature (RT). For 3D embedded cultures, the supernatant was carefully removed before filling the wells with 4% (w/v) PFA for 20 min at RT. After fixation, samples were washed with PBS and stained with Hematoxylin (Mayer’s, 1:1 Hematoxylin: PBS) for 10 sec in order to identify the spheroids in the paraffin blocks, and then washed three times in PBS. In order to preserve the 3D complex (co)-culture structure during the dehydration procedure and consecutive embedding in paraffin, a slightly modified protocol from Pinto *et al*.^24^ was used: Fixed spheroid pellets were suspended in 30 μl warm Histogel (Thermo Scientific HG-4000-012). For Matrigel/collagen and collagen embedded cultures, three 96-well gel plugs were extracted from the wells and sandwiched between two layers of Histogel in 8-well chamber slides (Labtek #155411). Cooled Histogel plugs containing the samples were dehydrated in 1 × 50% isopropanol (20 min), 2 × 70% isopropanol (20 min), 2 × 95% isopropanol (30 min) and 3 × 100% isopropanol (30 min) at 37 °C. At this point samples could be stored overnight in 100% isopropanol at 4 °C before further processing. For good infiltration of paraffin, samples underwent 3 changes with paraffin wax (each 30 min, at 65 °C) before paraffin embedding.

Alginate-BR samples were collected and fixed in 4% PFA (w/v)/4% sucrose (w/v) for 20 min at RT. As above, microencapsulated mono- and co-cultures were pre-stained with Hematoxylin for 10 sec and then washed three times with PBS. Cultures were pelleted and embedded in 1% (w/v) high melting temperature agarose (Lonza), dehydrated as above, and then embedded in paraffin wax.

#### TMA preparation from FFPE blocks, staining, and analysis

FFPE blocks are archived centrally at The Institute for Molecular Medicine Finland (FIMM) as tissue microarrays (TMA). TMAs were constructed by punching a single core with a diameter of 5 mm from the FFPE block using a semi-automatic punching device (MiniCore, Mitogen, UK). For FFPE samples with high cell density, a 1 mm core diameter was used. After arraying, TMAs were cut in 4 μm sections on Superfrost objective glasses (O. Kindler Gmbh, Germany) using Microm 355S microtome (Thermo Scientific, Waltham, MA). Slides were dried and stored at −20 °C.

For histopathological examination, paraffin sections (4 μm) of TMAs were stained with hematoxylin and eosin (H&E). IHC was carried out by a standard protocol (details in supplementary methods). Primary antibodies used were as follows: Ki67 (Abcam; EPR3610; #92742; 1:4000), cleaved caspase 3 (Cell Signaling Technology; #9661; 1:300), estrogen receptor (Abcam; SP1; #16660; 1:100), androgen receptor (Santa Cruz; #13062; 1:750), cleaved CK18 (Roche; M30 Cytodeath; #12140322001; 1:200), gamma H2AX (Cell Signaling 2577; 1:200), phospho-Histone H3 (Upstate 06–570; 1:1000), cytokeratin 8 (Abcam 53280; 1:2000), Vimentin(Dako M0725; 1:10000), and E-cadherin (Cell Signaling 3195; 1:1000). Stained TMAs were imaged at 0.33 μm/pixel resolution using a Pannoramic P250 Flash II whole slide scanner (3DHistech, Hungary) equipped with a Plan-Apochromat 20x (NA 0.8) objective (Zeiss, Germany). Scanned images were uploaded on a Webmicroscope platform, to which all PREDECT members had access (http://predect.webmicroscope.net/).

After downloading the images at a scale of 1:4 from the Webmicroscope using SpotFinder, they were analyzed using the cell counter plugin from imageJ^22^.

#### Statistical analysis

Data sets were analyzed statistically using GraphPad Prism 6, or R scripts for image analysis, and tested for normality using the Shapiro-Wilks test. Two-tailed significance tests were performed with p < 0.05 considered significant. Non-parametric analyses were done with the Mann-Whitney-U-Test, parametric with the t-test. For image analysis data, the extension by Welch for unequal variances was used along with a multivariate ANOVA for the comparison of proliferating cells and spheroid size. Multiple groups were compared using a one-way ANOVA using the Tukey post-hoc test. Significances are depicted as n.s.: not significant, *p < 0.05, **p < 0.01, ***p < 0.001.

## Results

### Generation of model platforms

**Figure 1:**
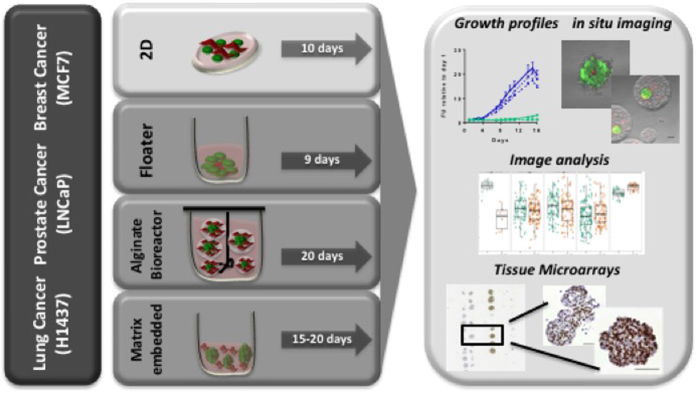
The PREDECT 2D/3D model workflow. Three cell lines (MCF7, LNCaP, H1437) belonging to three pathologies (breast, prostate, lung) were set up as 2D or 3D models in mono- and co-cultures. Growth was monitored via fluorescence, and the cultures imaged *in situ* by confocal microscopy and analyzed via dedicated 2D/3D imaging software, or fixed, paraffin embedded, and processed into TMAs and then analyzed via immunohistochemistry (IHC).

The goal of the PREDECT consortium is to characterize *in vitro* tumor models for target identification and validation by multi-modal cross-comparisons. Models described here include 2D and floating spheroid cultures (“floaters”), cultures embedded in alginate and grown in stirred-tank bioreactors (“alginate-BR”), as well as extracellular matrix (ECM) embedded cultures, all as mono- and co-cultures with stromal fibroblasts (Fig. 1). Cell lines and respective fluorescence tags are listed in Table 1, culture set up in Table 2.

### Characterization of model platforms using growth curves

Growth curves, based on fluorescence measurements, were established for all the models. The data set for the MCF7 cell line is described in more detail as a prototypic model, the results for the LNCaP and H1437 cell lines are summarized as supplementary information.

**Figure 2:**
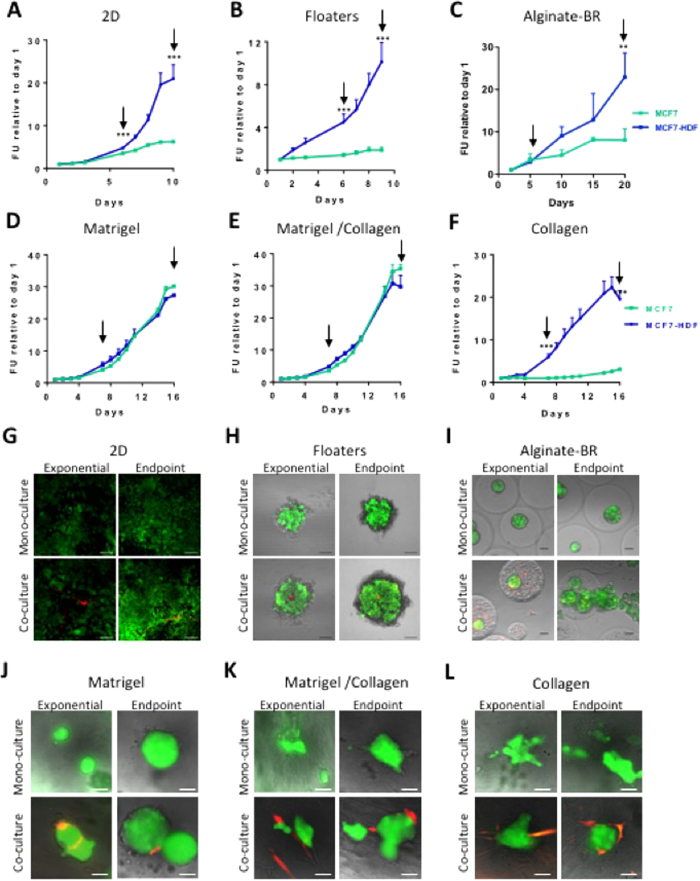
MCF7 breast cancer model characterization using fluorescence-based growth curves. Models were set up, and growth of MCF7 tumor cells was measured via GFP fluorescence as described in M&M. Stromal cell growth could not be detected. The graphs in the upper panels show growth curves of the mono- (green) and co-cultures (blue) grown in (**A**), 2D, (**B**), floaters, (**C**), alginate-BR, (**D**) Matrigel, (**E**) Matrigel/collagen, (**F**) collagen. Arrows indicate time-points when pictures were taken; significant differences between mono- and co-cultures are indicated (**p < 0.01; ***p < 0.001). In G-L, fluorescence images corresponding to (**A–F**) are shown. GFP-labeled tumor cells in green, RFP-labeled HDF stromal fibroblasts in red. Scale bars: 2D, floaters: 100 μm; alginate-BR, embedded: 50 μm.

Growth of MCF7 breast cancer cells in 2D cultures was stimulated by the presence of human dermal fibroblasts (HDFs) from day six on. Both mono- and co-cultures reached the stationary phase around day ten, but the co-cultures attained more than triple the signal of the mono-cultures (Fig. 2A).

Also in floaters, the presence of HDFs stimulated growth by about ten-fold (Fig. 2B). Co-cultures formed inside-out spheroids, with the fibroblasts nestled in the spheroid core (Fig. 2H, co-culture images; Fig. S1 for a higher magnification of the HDFs in the core of the spheroid).

For the alginate-BR cultures, MCF7 cells were aggregated for 24 hours and then microencapsulated in alginate in the presence or absence of HDFs as a single cell suspension, as described in ref. 11. This protocol aims to keep the two compartments in close proximity but prevents the formation of inside-out spheroids as observed in the floater cultures (Fig. 2I).

The presence of HDFs in the alginate microcapsules lead to a growth stimulation of the tumor cells compared to mono-cultures, which reached significance at day 20 (Fig. 2C). Interestingly, co-cultures presented an altered morphology and behavior: aggregates formed irregular structures that leaked out of the alginate microcapsules (Fig. 2I). This was rarely observed in MCF7 mono-cultures (Fig. 2I). HDFs deposit collagen and other ECM components in the alginate capsule, and also secrete pro-inflammatory factors^11^,^25^. Increased collagen density^26^, but also paracrine factors, *e.g*., the cytokine IL6^27^, or growth factors like SDF1 and FGF2, amongst others^28^, have been shown to drive growth and invasion of mammary tumor cells *in vitro* as well as in mouse models.

MCF7 growth as embedded mono-cultures was strongly matrix-dependent. Matrigel-containing matrices supported growth well, as shown by an approx. 30-fold fluorescence increase over time (Fig. 2D,E), whereas the tumor cells alone hardly grew in pure collagen (Fig. 2F). Admixing HDFs to the collagen-embedded cultures stimulated MCF7 cell growth approx. 6-fold, but fluorescence levels reached at stationary phase were still lower than observed in Matrigel-based cultures (Fig. 2D–F, please note different Y-axes). This confirmed observations made by Krauss and co-workers who described poor growth of MCF7 mono-cultures in collagen^29^. MCF7 cultures grown in Matrigel-containing matrices did not benefit from the addition of HDFs (Fig. 2D,E). In all matrices, fibroblasts were observed to closely contact the tumor spheroids (Fig. 2J–L).

To conclude, HDFs stimulated MCF7 growth in all culture formats, except for Matrigel-containing embedded models. Besides structural basement membrane constituents such as laminin, fibronectin, collagen IV *etc*., Matrigel (although growth factor depleted) still contains significant amounts of growth factors that may partially obfuscate the effect of the stromal cells^30^.

### Response of model platforms to standard of care (SOC) treatment

Targeted compounds, as well as a chemotherapeutic SOC treatment, were used to further characterize the models. For estrogen receptor positive breast cancer, Fulvestrant, a full estrogen receptor antagonist^31^, for prostate cancer, MDV-3100, a second-generation androgen receptor inhibitor^32^, and for lung cancer, GSK1059615, a dual PI3K/mTOR inhibitor^6^ were chosen as targeted treatments. Docetaxel, an anti-mitotic agent, was selected as a chemotherapeutic SOC agent for all three pathologies^33^.

In a first step, IC_50_ values (IC_50_s) were established by generating dose-response curves for all models, starting treatment once exponential cell growth was observed (see Table S2). In a second step, growth curves were established for a single drug concentration per model in order to characterize the effect of the stromal cells on the treatments on a cellular level. At the endpoint, when control cultures reached the stationary phase, samples were fixed *in situ* for fluorescence analysis, and formalin-fixed and paraffin embedded for IHC. Targeted drugs, which mainly act as cytostatics, were used at an IC_80_ concentration, whereas Docetaxel, as a cytotoxic agent, was used at an IC_50_ concentration, in order to obtain analyzable samples at the endpoint.

**Figure 3:**
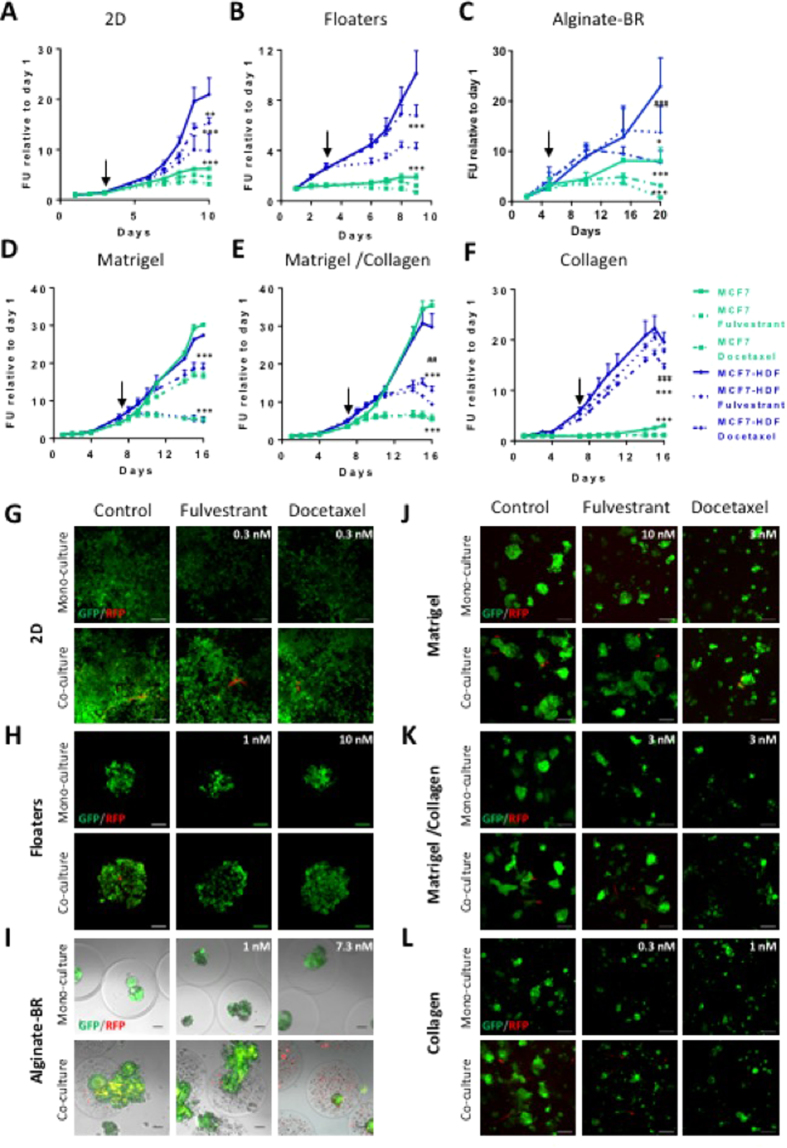
Fluorescence-based growth curves of MCF7 breast cancer models treated with Fulvestrant or Docetaxel. Models were set up, and growth of MCF7 tumor cells was measured via GFP fluorescence as described in M&M. Stromal cell growth could not be detected. Mono- and co-cultures were treated with Fulvestrant or Docetaxel at the concentrations indicated in the fluorescence images (**G–L**), corresponding to IC_80_ and IC_50_ values on mono-cultures. Untreated DMSO control curves are indicated with a straight, Fulvestrant with a dotted, and Docetaxel with a broken line. Shown are the graphs for mono-cultures (in green) or HDF co-cultures (in blue) grown in (**A**), 2D, (**B**), floaters, (**C**), alginate-BR, (**D**) Matrigel, (**E**) Matrigel/collagen, (**F**) collagen. Arrows indicate start of treatment. Significances are indicated for comparison to DMSO treated controls (*) or monocultures (#) (*p < 0.05; **p < 0.01; ***p < 0.001). In (**G–L**) fluorescence images corresponding to (**A–F**) are shown. GFP-labeled tumor cells in green, RFP-labeled HDF stromal fibroblasts in red. Scale bars: 2D and floaters: 100 μm; alginate-BR, embedded cultures: 50 μm.

Fulvestrant and Docetaxel affected MCF7 growth in 2D and floater cultures (Fig. 3A,B). The percent inhibition was comparable between mono and co-cultures, indicating that HDFs did not provide protection against either of the treatments (Fig. S2A/B). It should be noted, however, that Docetaxel and Fulvestrant treated co-cultures had higher GFP signals at the endpoint than untreated mono-cultures (Fig. 3A,B).

Docetaxel treatment decreased cell growth in alginate-BR mono- and co-cultures. In stark contrast, co-cultures appeared refractory to Fulvestrant treatment until day 16 (Fig. 3C). The latter cultures were indistinguishable from controls, showing a loss of spheroid roundness, and leakage from the alginate microcapsules (Fig. 3I). HDFs thus protected MCF7 from Fulvestrant in this culture format (Fig. S2C).

High-energy metabolites released by stromal fibroblasts may induce tumor cell mitochondria activity to protect from Fulvestrant^34^. Also, IL-6 secretion has been shown to confer estrogen-independence to MCF7 and other estrogen-dependent breast cancer cell lines^27^. Corroborating the latter, slightly increased levels of IL6 and IL8 were found in alginate-BR:HDF co-cultures^11^.

In the matrix-embedded 3D format, Docetaxel and Fulvestrant reduced MCF7 growth in all three matrices tested (Fig. 3D–F). Co-cultures provided only a minimal growth benefit in Matrigel, whereas in the Matrigel/collagen mix and collagen alone, the fibroblast co-cultures protected the tumor cells from treatment (Fig. S2D–F). Fibroblasts (in red) remained viable in Fulvestrant-treated co-cultures, whereas chemotherapy eradicated the fibroblasts except for when grown in Matrigel (Fig. 3J–L).

Thus HDFs provided not only a growth advantage, as described in the previous section, but also a treatment protection to MCF7 cells, mainly in culture formats with lower levels of nutrients and growth factors (i.e. alginate-BR and collagen).

### Image analysis of 3D cultures

In order to validate data from the growth curves, and to gain additional information on the characteristics of the models, fluorescence images were taken from cultures *in situ* and subjected to analysis by dedicated image software developed within the consortium^23^. Size, numbers, and proliferation rates of tumor spheroids in 3D cultures were measured, and correlated with the presence or absence of stromal cells, SOC treatment, and culture format. In order to adequately compare treated with untreated cultures, images taken at the end of the culture period were analyzed.

**Figure 4:**
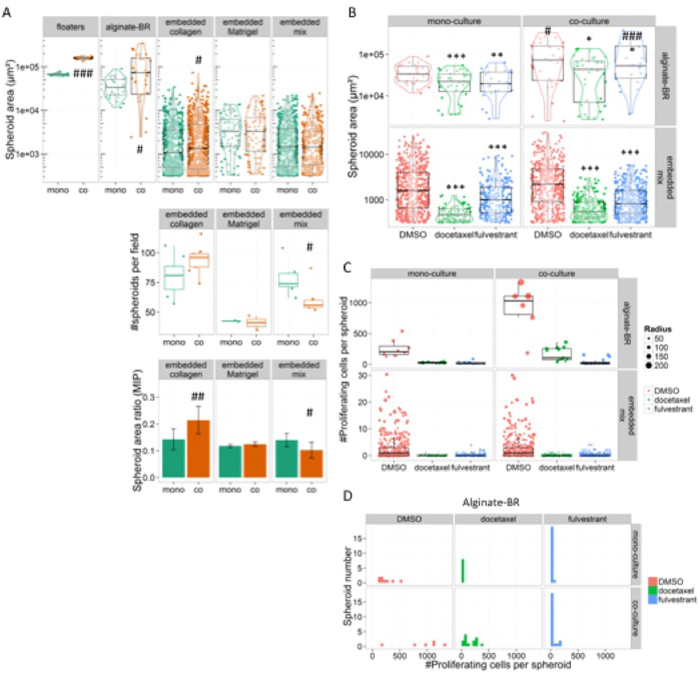
*In situ* image analysis & quantification of 3D models. (**A**) Scatter plots comparing MCF7 3D models by spheroid sizes (upper graph) and numbers (lower graph). Mono- (green dots) and HDF co-culture (orange) spheroid areas and numbers derived from maximal intensity projection (MIP) analyses are displayed. The size analysis is shown on a logarithmic scale (upper graph). (**B**) Scatter plots comparing unperturbed (red) and Docetaxel (green) or Fulvestrant (blue) treated MCF7 3D cultures via spheroid areas, displayed on a logarithmic scale. (**C**) Quantification of EdU-positive cells in mixed matrix embedded or alginate-BR MCF7 mono- and HDF co-cultures, treated with Docetaxel (green), fulvestrant (blue) and DMSO control (red). Scatter plots of EdU-positive cells per spheroid, with the size of the dots corresponding to the radii of the spheroids, are displayed. Negative spheroids are collapsed to the bottom of the plot. (**D**) the alginate-BR data of (**C**) is shown as a histogram indicating the number of proliferating cells per spheroid. Note the increase of EdU-positive spheroids in the HDF co-cultures, and the large number of non-proliferating spheroids in Fulvestrant treated cultures. P-values for the comparison between treatment conditions are represented by *-symbols (*p< 0.05; **p<0.01; ***p<0.001), while #-symbols are used for comparison between mono- and co-cultures (^#^p < 0.05; ^##^p < 0.01; ^###^p < 0.001).

Size analysis revealed a characteristic mean and spread for each of the 3D culture formats. Floater cultures formed the largest spheroids, with a very small size distribution (0.066 ± 0.004 mm^2^), and a large gain induced by HDF co-cultures (0.16 ± 0.01 mm^2^). Alginate-BR spheroids were next in size, but showed a wider spread size distribution (0.10 ± 0.09 mm^2^ for co-, and 0.037 ± 0.018 mm^2^ for mono-cultures; Fig. 4A). Matrix-embedded spheroids were clearly smaller than the previous two models, ranging between 0.001–0.003 mm^2^. Amongst the latter, Matrigel supported the largest, and collagen the smallest spheroids, corroborating observations from growth curves (Figs 2D–F and 4A). The strong effect of the HDFs on collagen-embedded samples observed in the growth curves (Fig. 2F) translated into significantly increased average spheroid areas, although the difference was not as striking as observed in the growth curves (Fig. 4A, upper panel). When counting spheroids per area, we found that HDFs also affected MCF7 spheroid numbers (Fig. 4A, lower panel). As a result, the total spheroid density is higher, as illustrated by the spheroid foreground area fraction in Fig. 4A, lower panel.

Docetaxel and Fulvestrant treatment reduced spheroid sizes of alginate-BR and embedded samples, confirming the results from fluorescence-based growth curves (Fig. 4B).

Analysis of tumor spheroid size and numbers *in situ* represents a relatively simple imaging tool that may be used to validate and complement simple fluorescence growth analysis. The results from the growth curve analyses shown in the previous sections were largely confirmed, validating the approach of using fluorescence as a proxy for cell culture growth.

In order to measure effects of the culture formats or SOC treatment on proliferation, alginate-BR and embedded samples were incubated with EdU for 2 hours before fixation to label cells with ongoing DNA synthesis. EdU-positive cells were counted per spheroid using an algorithm developed to analyze spheroids in 3D^23^.

For both alginate-BR, and mixed matrix embedded cultures, a clear treatment-dependent reduction of EdU positive nuclei/spheroid was detected (Fig. 4C, compare DMSO (red) with Docetaxel (green) and Fulvestrant (blue) treated cultures). The growth-stimulatory effect of admixing HDFs to MCF7 alginate-BR cultures was also evident by the increase of EdU-positive nuclei per spheroid and the concomitant boost of the average spheroid size (Fig. 4C). In Docetaxel treated samples, the presence of HDFs in MCF7 alginate-BR cultures sustained some EdU-positive nuclei, whereas Fulvestrant imposed a complete proliferation block, as suggested by the total lack of EdU-positive cells (Fig. 4C). The histogram representation of the above results reveals the large number of non-proliferative spheroids in the Fulvestrant treated cultures, which amassed at the baseline in the previous scatter plot (Fig. 4E). Fulvestrant thus imposed a relatively slow-acting proliferation block, leaving the spheroids mostly intact, whereas Docetaxel killed proliferating cells, resulting in the dissolution of the spheroids. In conclusion, both treatments were ultimately active against alginate-BR cultures, but the timing and the effect on the cells (cytostatic versus cytotoxic) varied, explaining the differential effect on the growth curves in the bioreactor (Fig. 3C).

### Analysis of tissue microarrays (TMA)

In order to enable the PREDECT consortium to perform a side-by-side comparison of models and patient samples, protocols were developed that allowed for fixing, adequate paraffin embedding and staining of the samples in TMAs. The 3D conformation of all model formats could be preserved through paraffin-embedding, except for Matrigel, which dissolved during fixation.

The TMAs were stained for a number of markers in order to characterize cell types and cell cycle status (cytokeratin 8 (CK8), E-cadherin, estrogen receptor (ER), androgen receptor (AR), Vimentin, Ki67, phospho-Histone H3 (pHH3), cleaved cytokeratin 18 (cCK18), gamma H2AX, cleaved caspase 3 (cC3)).

**Figure 5:**
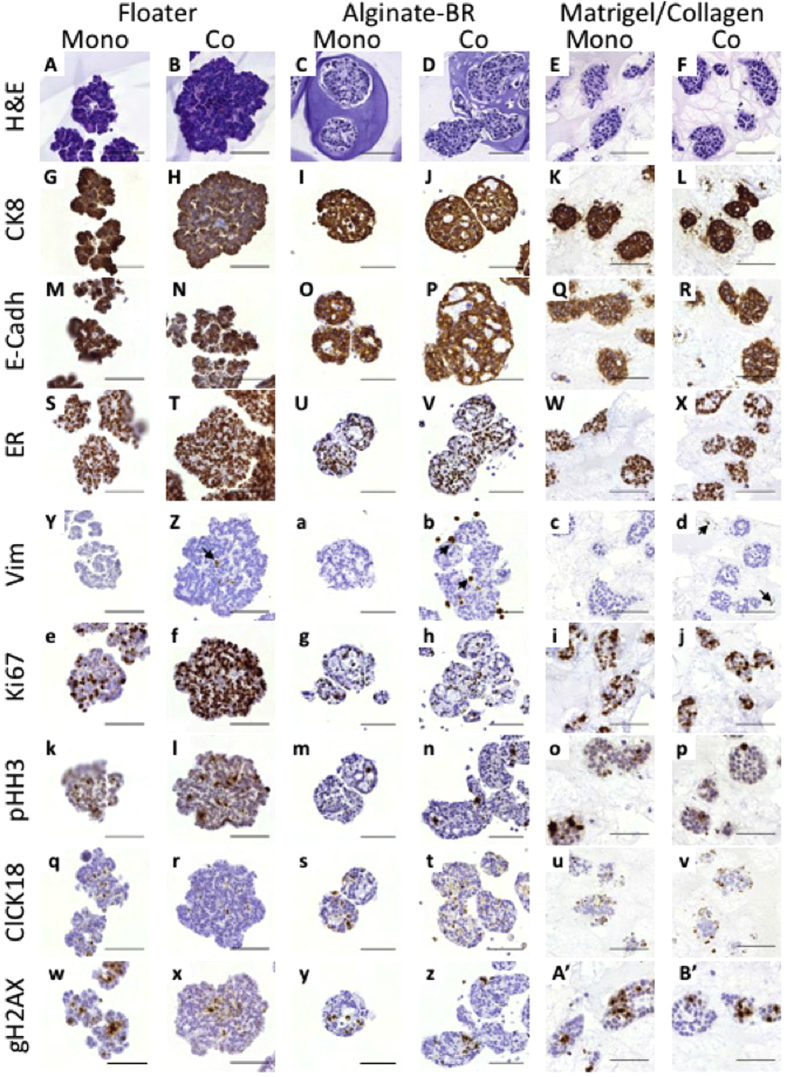
IHC analysis of breast samples. Cultures were fixed, paraffin embedded, and arrayed into TMAs as described in Materials and Methods. TMAs were then stained H&E (**A–F**), as well as for cytokeratin 8 (**G–L**), E-cadherin (**M–R**), estrogen receptor (**S–X**), Vimentin (**Y–d**), Ki67 (**e–j**), phospho-Histone H3 (**k–p**), cleaved CK18 (**q–v**), and gamma H2AX (**w–B**′), respectively. Antibodies were detected using a chromogenic DAB assay (brown), and samples were counterstained with hematoxylin (blue). Size bars indicate 100 μm.

H&E staining of the MCF7 models showed the characteristic 3D morphology of the spheroids grown in the various culture formats, and revealed differences in their cellular organization, *e.g*., while floater spheroids were rather compact, alginate-BR spheroids displayed small lumens inside (Fig. 5A–F).

CK8 and E-Cadherin as epithelial cell markers were expressed on MCF7 tumor cells in all models (Fig. 5G–R). As expected, MCF7 cells expressed ER in the nucleus, although some negative cells were found in the alginate-BR, indicating model-induced heterogeneity (Fig. 5S–X). Vimentin-positive fibroblasts were found in all co-culture models (indicated by arrows in Fig. 5Z,b,d). As suggested by the fluorescence images, fibroblasts localized to the core of the floater cultures, forming inside-out spheroids (Fig. 5Z). Whereas most of the fibroblasts in the alginate-BR format were found outside of the spheroids, some had invaded the spheroid core (Fig. 5b). This was not observed in embedded cultures (Fig. 5d).

Proliferation was assessed with the general proliferation marker Ki67 and the M-phase-specific marker phospho-Histone H3 (Fig. 5e–p). HDF co-cultivation in floater cultures resulted in enhanced proliferation compared to mono-cultures (Ki67: 20.9 ± 1.8% vs. 84.8 ± 1.7% ***p; pHH3: 1.9 ± 0.4% vs. 6.2 ± 1.4% **p). In alginate-BR mono- and co-cultures, approximately 40% or 10% of MCF7 cells were Ki67 and pHH3 positive, respectively. No significant differences were observed between mono- and co-cultures, which also held true for embedded samples.

Unfortunately, despite employing IC_50_ or IC_80_ concentrations rather than maximally efficacious doses for Docetaxel and targeted therapies, respectively, treated samples proved very fragile and unstable, and many were lost during processing, rendering analysis of treatment effects by TMAs rather impractical.

In conclusion, protocols have been established for *in vitro* tumor models with increasing complexity. They were validated using exemplary cell lines for three pathologies, breast (MCF7), prostate (LNCaP), and lung (H1437), following growth and response to SOC treatment via fluorescence-based growth curves. These results were corroborated and complemented by *in situ* 3D image analysis, as well as IHC on TMAs derived from most models. Our results demonstrate that in order to more faithfully evaluate the relative contributions of co-cultured cells or matrix composition on baseline tumor cell growth rates or responses to drug treatments, a multi-pronged approach to the analysis and set-up of complex 3D assays is required. Table S3 provides a comprehensive overview on the results of our study, including a rough cost estimate for the set-up of a 96-well plate, spheroid sizes obtained in each of the model, fold growth over the course of the experiment, and the effects of the set-up on tumor drug sensitivity.

## Discussion

The goal of the present study was to establish robust protocols for 2D and 3D culture methods and thoroughly characterize them via side-by-side comparisons in order to better understand their applicability for oncology drug discovery. The outcome showed that even with a relatively small matrix of tumor and stromal cells in a set of different culture formats, the interactions of the individual components result in altered growth characteristics or drug responses of the cultures. Thus none of the models presented here will be able to capture all of the aspects of tumor biology, but they differ in the degree to which they represent such aspects, and should be used accordingly. Along the same line, while some of the observations from this study will be cell type specific, others are more related to model set-up, and may therefore be used to draw more generally applicable conclusions. Below, we will try to disentangle these observations, and come up with some recommendations that should help to set up complex models in a robust and informative manner for drug discovery and validation purposes.

As shown in the Results section, appropriate analysis of the data from complex 3D models is of utmost importance. While fluorescence based growth curves are simple and applicable to relatively high throughput experiments, crucial information may be lost by looking at an average response of a whole culture set-up. In the first section of the Discussion, the approaches to analyze complex 3D models presented in this study will be compared with respect to their cost and benefit.

Since potentially very labor- and time consuming, complex image analyses should strictly focus on key questions. Here, image analysis was used mainly for two questions, (1) is the approach of using fluorescence intensity measurements valid as a proxy for tumor cell growth? and, (2) can we define effects of the culture format and the treatments more precisely by molecular imaging, in this case using EdU as a label for replicating cells?

Requirements on image quality (and hence also equipment) depend on the analyses to be performed. Number, size, and shape analyses are less demanding than molecular imaging. Sample number and related microscope and computing time were the main issues, requiring the availability of an automated imaging system. In the present study, spheroid size analyses generally confirmed the data obtained from fluorescence growth curves, thus fulfilling its pre-defined task. Discrepancies between fluorescence growth curves and size analyses were found when spheroid size wasn’t the main differentiator between treated and untreated cultures (*e.g*. Fig. 4A, HDF effect on collagen-embedded MCF7 cultures), or when size did not correspond to the number of viable cells (*e.g*. Fig. S12A,B). Shape analysis, which may contribute further relevant information, *e.g*. invasiveness of 3D cultures^35^, was also performed, but did not provide much additional insight for this study.

Analysis of the proliferation index of the spheroids by quantifying EdU-stained nuclei proved a much more difficult task, despite the availability of a ‘2.5D analysis’ workflow that reduced the complexity of real 3D analysis to a combination of maximal intensity projection and height mapping^23^. Image quality had to be adequate: lateral and axial resolution as well as inter-slice distance had to be well below cell-radius (estimated 5 μm), and spheroids needed to be physically separated by at least 10 μm along the axial direction, and non-overlapping in the lateral direction. Moreover, spheroids larger than 100 μm could not be analyzed because of light attenuation. However, despite being resource-intense and demanding on image quality, the analyses performed for the present manuscript provided relevant information. The pro-proliferative effect of the HDFs on MCF7 could be shown on a cellular level, and the anti-proliferative effect of Fulvestrant as opposed to the cell killing mediated by Docetaxel could clearly be distinguished (Fig. 4C,D). Thus, if probes are available to monitor the effect of a given treatment by molecular imaging, the investment in molecular image analysis may well be worthwhile.

TMA-based analysis was chosen by the PREDECT consortium as the means of choice for the cross-comparison of models, and ultimately patient material, since tissue heterogeneity is preserved, and it represents the gold standard for pathologists.

Processing samples for TMAs proved labor intensive, and was not entirely successful, *e.g*. analysis of treated samples was severely hampered by the loss of already treatment-reduced sample material. However, successful analyses addressed several open questions, or independently confirmed previous findings. These included the presence of stromal cells in lung and breast floaters (Figs 5 and S13), the puzzling apparent lack of an effect of Docetaxel on H1437 alginate-BR spheroids (Fig. S12), and the increase of fibroblasts in treated LNCaP models (Fig. S11B). Moreover, avenues for further investigations were opened, such as the suggestion of an EMT induction in LNCaP 3D samples (Fig. S11C). The latter represents a good example for the necessity to preserve sample heterogeneity, even in LNCaP mono-cultures. A more in-depth analysis of the results, and their comparison to other models and patient samples, remains yet to be done, and will be published in a follow-up manuscript by the PREDECT consortium. Thus albeit labor intensive, once samples have been generated and arrayed into TMAs, the depth of analysis possible is only restricted by the number of antibodies that work in FFPE material.

The next sections focus on ‘lessons learnt’ from the analysis of the models presented here. As mentioned above, some observations appeared model/set-up specific, and are therefore likely to translate to additional cell systems or pathologies, whereas others were clearly cell-type-specific, and will need to be established for every model. However, also for the latter, some general guidelines that may help to speed up the set-up time for new models will be discussed.

Spheroid sizes appeared to be platform rather than pathology-dependent. Spheroid size matters, since only when a minimal radius of about 200 μm is exceeded, spheroids may form apoptotic cores due to hypoxia and lacking nutrients^36^,^37^,^38^. Hypoxia is prevalent in human tumors due to lacking, or disorganized vasculature, and numerous approaches have been explored to target hypoxia as a treatment paradigm^39^. Another aspect of spheroid size is drug penetration. Spheroid cultures have been shown to be resistant to cytotoxic agents due to a lack of appropriate drug exposure^40^,^41^,^42^. For some drugs, this could be directly correlated to spheroid sizes^43^. Ineffective drug penetration into solid tumors indeed has been suggested to lead to drug resistance^44^. Not surprisingly, also gene transcription is strongly affected by spheroid sizes (Boghaert *et al*., manuscript in preparation). Larger LNCaP floaters had apoptotic cores (Fig. S11A), and the cultures showed some resistance to MDV-3100, albeit not Docetaxel (Fig. S6).

Differences between Matrigel and Collagen embedded cultures have been observed consistently across the models. Overall, cultures containing Matrigel tended to grow better, or be less sensitive to growth inhibition, or both. (1) MCF7 cells grew well in Matrigel or mixed matrix, but collagen was a poor substrate; (2) the maximal growth inhibitory effect of MDV-3100 on LNCaP cultures in Matrigel containing matrices was reduced to about 30–40% (compared to >60% in collagen; data not shown); (3) the IC_50_ values for Docetaxel and GSK1059615 treated H1437 cultures were approx. six fold higher in pure Matrigel than in collagen (Table S2). Growth factors present in the Matrigel preparation (even if growth-factor reduced) may provide cues for growth, survival, and invasion of the tumor cells, although the differential composition of the extracellular matrices may also play an important role. Various Matrigel lots may behave differently, which is why performance tests are imperative, and all groups working on this manuscript used the same Matrigel batch. Attempts to use synthetic Matrigel replacement matrices have not proven satisfactory yet in our hands (data not shown), but if successful, such matrices might provide the means to disentangle matrix from growth factor-dependent effects.

Growth factors and/or cues from basement membrane components within Matrigel also appeared to override aspects of co-cultures. Breast MCF7 cultures responded with a growth boost when incubated with HDFs in all but Matrigel-containing culture formats (Fig. 2). Also, the protective effect of HDFs was much more pronounced in collagen than in Matrigel-containing culture formats (Fig. S5). Similarly, CAFs increased the IC_50_ of GSK1059615 on H1437 in collagen by >30-fold. This was less pronounced in mixed matrix cultures, and not detected in Matrigel (Table S2). Thus, this would be consistent with a certain Matrigel-induced override of the communication between co-cultured stromal cells and tumor cells. Interestingly, however, the effect of co-cultures may even surpass the Matrigel effect, as was the case for GSK1059615 treated H1437 cells in co-cultures with CAFs (Table S2). But then, MCF7 cells in collagen failed to reach the fold fluorescence increase as in Matrigel, even in the presence of HDFs.

How important is the correct source of stromal fibroblasts? In prostate and lung, immortalized tissue-specific CAFs were available to the consortium, but not for the breast models, despite efforts to cultivate and immortalize primary fibroblasts from patients. HDFs were used as a substitute, but a sample of CAFs was purchased from a commercial vendor, and compared to HDFs in the spheroid model. A comparable growth stimulation was found for both types of fibroblasts (Fig. S14A), suggesting functional adequacy of the HDFs, as also observed by other groups^14^. In the lung H1437 alginate-BR model, HDFs provided a growth stimulation of the tumor cells, but not CAFs or NFs (Fig. S5B). Thus, although it may have to be confirmed on a case-to-case basis, HDFs appear to represent a viable substitute for tumor-specific CAFs, eliminating the need for difficult-to-obtain CAFs from cancer patients, which may prevent most non-specialized labs from their routine use.

No one single tumor:stroma ratio could be identified that would be optimal for all models. For MCF7 cultures, a strong growth-promoting effect by HDFs was observed in most culture formats, across ratios of 1:1 to 10:1. In the lung, a 10:1 ratio did not provide a growth benefit in 2D, whereas a 1:1 ratio did (Fig. S4A vs S5A), suggesting a slightly less flexible system. However, such an initial tumor:stroma ratio is not expected to remain constant over the course of an experiment. For example, in the MCF7/HDF alginate-BR co-culture model, the original 1:1 ratio shifted in the course of the experiment to approximately 6:1, mainly due to a lack of stromal cell proliferation (data not shown). In the floater cultures, the original 3:1 ratio likely increased even more dramatically over time, since at the end of the experiment only very few stromal cells were left at the center of the tumor (Fig. 5). Only in treated prostate tumor LNCaP/WPMY-1 co-culture models was a decrease of the tumor:stroma ratio over time observed (Fig. S6). In an experiment to check the optimal tumor:stroma ratio for collagen embedded MCF7 cultures, a range of ratios between 1:1 to 20:1 was tested. Only at a ratio of 20:1 dropped the growth rate down to near mono-culture levels (Fig. S14B). Thus, even though human pathology should be used as a rough guideline for a given pathology, a range of tumor:stroma ratios between 1:1 to 10:1 appears to be a good starting point for a co-culture set-up.

In conclusion, there is no one-fits-all model, the choice of the model depends entirely on the question that needs to be answered (see Table 3). If the genetic make-up of a tumor cell that responds to a given treatment needs to be found, then a screen of a large number of molecularly characterized cell lines in 2D may be sufficient^45^. However, if a given target relies on cell-cell, or cell-ECM interactions, more complex models will be required. This is also true if not only sensitivity, but also resistance to a given new treatment should be investigated. Thus, for studies, which consider drug penetration issues, or target hypoxia (*e.g*. ref. 19), floater spheroid cultures appear best suited since appropriate spheroid sizes are reached. Alternatively, pre-grown larger spheroids embedded in ECM, hypoxia chambers, or the alginate-BR system in low O_2_ conditions may be applied for such studies. When looking at the effect of the ECM, Matrigel embedded cultures are most widely used. However, results may be confounded by the presence of growth factors and their concentration heterogeneity in different batches. Therefore, collagen or, once shown to be a real alternative, synthetic matrices in the presence or absence of stromal co-cultures may be a better choice^46^,^47^. Also alginate-BR cultures, which provide a biologically inert structure that may be functionalized by the encapsulated cells, appear to be a viable alternative. Tumor:stroma ratios between 1:1 and 10:1 were found to be functional, with lower ratios to be used in systems where fibroblast growth is expected to be minimal, resulting in decreased, more physiologically relevant ratios in ‘mature’ cultures. This should also be considered when looking at compounds that specifically target the interface between tumor and stromal cells^48^. If looking at compounds that target stroma for resistance mechanisms, a small matrix of tumor and stroma cells should be tested in order to best cover tumor heterogeneity, as has already been performed in 2D^49^. Also, not only stromal cells but also macrophages and other constituents of the immune system, as well as components of the vasculature play important roles in treatment resistance and should be included in *in vitro* assays that may ultimately get closer to model patient tumors^50^,^51^,^52^.

## Additional Information

**How to cite this article**: Stock, K. *et al*. Capturing tumor complexity *in vitro*: Comparative analysis of 2D and 3D tumor models for drug discovery. *Sci. Rep*. **6**, 28951; doi: 10.1038/srep28951 (2016).

## Supplementary Material

Supplementary Information

## Figures and Tables

**Table 1:** Cell lines, type, origin, label.

Cell line (source)	Type	Origin	Label
**MCF7** (ATCC HTB-22)	Epithelial	Breast Adenocarcinoma	eGFP-FLuc2
**HDF** (Innoprot P10857)	Fibroblast	Skin	tagRFP
**LNCaP** (ATCC CRL-1740)	Epithelial	Prostate Adenocarcinoma	tRFP-FLuc
**WPMY-1** (ATCC CRL-2854)	Myofibroblast	Prostate	eGFP-RLuc
**CAF- PF179T^1^** (ref. 8)	Fibroblast	Prostate Adenocarcinoma	eGFP-RLuc
**NCI-H1437^2^** (ATCC CRL-5872)	Epithelial	Lung Adenocarcinoma	tdTomato
**CAF3**[Fn t1-fn3]	Fibroblast	Lung Adenocarcinoma	eGFP
**NF^3^**	Fibroblast	Lung	eGFP

^1^Referred to as CAF.

^2^Referred to as H1437.

^3^As described in M&M.

**Table 2:** Experimental cell culture conditions.

	2D			3D floater			3D matrix			3D bioreactor			Medium
	Tumor cells^1^	Tumor: stroma	FBS (%)	Tumor cells^1^	Tumor: stroma	FBS (%)	Tumor cells^1^	Tumor: stroma	FBS (%)	Tumor cells^1^	Tumor: stroma	FBS (%)	
Breast	6,000	3:1	10	1,000	3:1	10	10,000	10:1	2	20000/ml	1:1	10	DMEM
Prostate^2^	10,000	10:1	2	1,000	10:1	2	10,000	10:1^3^	2	NA			RPMI
Lung	10,000	10:1	10	400/200^4^	1:1	10	10,000	10:1	2	20000/ml	1:1	10	RPMI

^1^Stromal cells were added on top (except for lung in 3D floater cultures).

^2^2D and 3D spheroids contained 0.01 nM R1881 (Methyltrienolone).

^3^Inclusion of more than 1000 fibroblasts resulted in collagen contraction after less than a week.

^4^400 tumor cells for mono-cultures, 200 for co-cultures.

**Table 3:** Overview of platforms with strengths/weaknesses & suggested applications.

Model		Plus	Minus	Applications
2D	mono	- simple, cheap- reproducible- fully automatable-H/MTS amenable	- limited predictivity for drug effects in patients- extremely high matrix stiffness	- (TD) tumor cell signaling- (TD, R) genetic alterations in tumor cells
	co	- *stroma derived growth benefit/drug resistance*	- space restriction	- *(TD, R) tumor-stroma interactions*
floater	mono	- *3D architecture*- drug penetration through tumor cell layers- hypoxic core (if large enough)- fully automatable - MTS amenable	- *not all tumor cells form spheroids*- *requires assaydevelopment (uniformity, growth…)*- *complex assay analysis*- no extracellular matrix interaction	- *(TD, R) 3D architecture*- (TD, R) tumor hypoxia- (R) drug penetration
	co		- often form inside-out spheroids- very few stromal cells survive	
BR	mono	- control of physico-chemical parameters- large batches of uniformly encapsulated spheroids - tumor-specific ECM deposition	- re-plating of spheroids for multi-parameter testing - initial investment for set-up	- (TD, R) tumor-specific ECM deposition
	co	- preformation prevents inside-out spheroids - stromal cells provide GF and ECM		- (TD, R) stromal GF, ECM deposition
matrigel	mono	- provides rich basement membrane-like matrix- widely used, well published	- contains variable amounts of GF- considerable batch-to-batch variations- melts upon fixation in formalin	- *(TD, R) extracellular matrix*- *(TD) invasion*- (R) matrix-derived GF
	co		- fibroblasts do not differentiate- co-culture effects may be masked by matrix GF	
collagen	mono	- provides interstitial stroma matrix environment- simulates invasive environment	- some epithelial tumor cells may not grow	
	co	- natural environment for fibroblasts- no/low confounding GF present	- activated fibroblasts contract matrix	- (TD, R) stromal GF

Italic: apply for methods below as well (tumor-stroma effects only for co-culture models);

Abbreviations: H/MTS: high/medium throughput screening; GF: growth factor(s); ECM: extracellular matrix; (TD): find targets/test drugs addressing; (R): resistance mechanisms related to.
