# Supplementary material for: Capturing tumor complexity in vitro: Comparative analysis of 2D and 3D tumor models for drug discovery
Source: Sci Rep. 2016 Jul 1;6:28951. doi: 10.1038/srep28951 (PMC4929472; doi:10.1038/srep28951)
Supplement: Supplementary Information [file srep28951-s1.doc]

## Capturing tumor complexity *in vitro*: Comparative analysis of 2D and 3D tumor models for drug discovery

## Supplementary information

Kristin Stock^1#^, Marta F. Estrada^2,3#^, Suzana Vidic^4,5,6#^; Kjersti Gjerde^4,5#^; Albin Rudisch^7#^; Vítor E. Santo^2,3#^; Michaël Barbier^4^, Sami Blom^9^, Sharath C. Arundkar^10^; Irwin Selvam^7^; Annika Osswald^7, 11^; Yan Stein^10^, Sylvia Gruenewald^1^; Catarina Brito^2,3^; Wytske van Weerden^5^; Varda Rotter^10^; Erwin Boghaert^8^; Moshe Oren^10^; Wolfgang Sommergruber^7^; Yolanda Chong^4^; Ronald de Hoogt^4^, Ralph Graeser^12*^

^1^Bayer Pharma AG, Muellerstr. 178, 13353 Berlin, Germany

^2^iBET, Instituto de Biologia Experimental e Tecnológica, Apartado 12, 2780-901 Oeiras, Portugal

^3^Instituto de Tecnologia Química e Biológica António Xavier, Universidade Nova de Lisboa, Av. da República, 2780-157 Oeiras, Portugal

^4^Janssen Pharmaceutica nv, Turnhoutseweg 30, 2340 Beerse, Belgium

^5^ERASMUS MC, Wytemaweg 80, 3015 CN Rotterdam, The Netherlands

^6^Faculty of Mathematics, Natural Sciences and Information Technologies, University of Primorska, Koper, Slovenia

^7^Boehringer Ingelheim RCV, GmbH & Co. KG, Doktor-Boehringer-Gasse 5-11, 1120 Wien, Austria

^8^AbbVie, 1 North Waukegan Road, North Chicago, IL 60064-6098, USA

^9^Institute for Molecular Medicine Finland FIMM, Tukholmankatu 8, 00014 University of Helsinki, Finland

^10^The Weizmann Institute, 234 Herzl St., Rehovot 7610001, Israel

^11^Institute of Medical Genetics, Medical University of Vienna, Waehringerstrasse 10, A-1090 Vienna, Austria

^12^Boehringer Ingelheim Pharma GmbH & Co. KG, Birkendorfer Str. 65, 88400 Biberach an der Riß, Germany

^#^these authors contributed equally to this work

*email address corresponding author: ralph.graeser@boehringer-ingelheim.com

### Characterization of model platforms using growth curves

***LNCaP prostate and H1437 lung cancer cells***

Cell growth of both tumor cell lines, as evidenced by an increase of the fluorescence signal over time, was observed in all models (Fig. S3/4).

In prostate models, GFP-labeled WPMY-1, but not CAF, stromal cells also grew as mono-cultures (except for floaters, data not shown; Fig S3). No effect of stromal cell co-cultivation was observed on the LNCaP tumor cells. However, growth of WPMY-1 cells co-cultivated with LNCaPs in 2D or 3D embedded cultures appeared to be restricted, reaching a plateau much earlier than when grown as mono-cultures (Fig. S3). Space restriction in 2D, and/or growth factor depletion may explain this phenomenon, but tumor cells may also contribute to the control of stromal cell proliferation.

Co-culture with lung NFs and CAFs at a tumor:stroma ratio of 10:1 did not affect H1437 lung tumor cell growth in 2D, or embedded cultures, although a transient effect was observed in collagen and mixed matrix embedded cultures (Fig. S4A, D-F). In 2D, when the ratio of tumor cell:fibroblast was decreased to 1:1, however, tumor cell growth was stimulated (Fig. S5A). A ratio of 1:1 between tumor cells and fibroblasts also resulted in a growth stimulation of floater cultures (Fig. S4B). In the alginate-BR cultures, H1437 cell growth was not significantly affected by the presence of NFs or CAFs, even at a 1:1 ratio. However, HDFs at the same ratio enhanced growth of the tumor cells (Fig. S5B), albeit less striking than in the MCF7 breast model (Fig. 2A-C, F).

Whereas no benefit of stromal cell co-cultivation was observed in the LNCaP prostate models, growth of the lung H1437 cell line could be stimulated by stromal cell co-cultures, depending on the model, the tumor:stromal cell ratio, and the type of the stromal cells.

### Response of model platforms to SOC treatment

***Response of LNCaP prostate cancer models on SOC treatment***

The cell line selected for prostate, LNCaP, has a mutated AR with a broad steroid binding specificity, but responds well to anti-androgens ^1^. Indeed, all culture formats, with the notable exception of the 3D floaters, responded to the MDV-3100 (Fig. S6), with IC_50_s in the range of 10-100 nM (Table S2). All LNCaP culture models tested were sensitive to Docetaxel (Fig. S6), with IC_50_s between 1 and 20 nM (Table S2).

No effect of the stromal cells on the survival of LNCaP cells in 2D and 3D co-culture models was observed. However, whereas MDV-3100 or Docetaxel dose-response curves for LNCaPs cells showed a clear dose-dependent decrease of the RFP signal in 2D and embedded cultures, the WPMY-1 cell–derived GFP signal strongly increased with either treatment (Fig S7A). This was not observed in WPMY-1 monocultures (data not shown). With MDV-3100, WPMY-1 GFP fluorescence stayed high even at higher drug concentrations, suggesting that the cells were not dependent on androgen signaling for their growth. WPMY-1 express low levels of AR, but only a weak growth stimulation of the cells by androgens has been reported ^2^. Other than MDV-3100, Docetaxel at higher levels eliminated the WPMY-1 myofibroblasts. However, the delayed toxicity observed suggests that the myofibroblasts were approximately 1-2 logs less sensitive than the tumor cells (Fig S7A). An expansion of fibroblasts was also observed in images of the respective cultures collapsed to two dimensions (Fig. S7A; in untreated control cultures fibroblasts were very rare (data not shown)).

The observation of the WPMY-1 myofibroblast outgrowth upon treatment-induced tumor cell killing demonstrates the value of individually monitoring tumor and stromal compartments. A similar expansion of fibroblasts has been reported for colorectal cancer patients after chemotherapy ^3^, indicating that this observation may have clinical relevance.

***Response of H1437 lung cancer models on SOC treatment***

Docetaxel reduced growth of the H1437 cultures in all of the formats tested (Fig. S8). Sensitivity to the pan-PI3 kinase/mTOR inhibitor GSK1059615, however, depended on the culture format.

In 2D co-cultures, fibroblasts sensitized H1437 cells to GSK1059615 at a tumor:stroma ratio of 1:1, but not 10:1, possibly due to the enhanced proliferation rate (Fig. S8, S9). In embedded H1437 models, a complex picture of GSK1059615 sensitivity and resistance emerged. Collagen and mixed matrix mono- and NF co-cultures were very sensitive, with IC_50_s comparable to 2D and floaters (20-60 nM). Pure Matrigel appeared to provide some protection against GSK1059615, raising IC_50_s to 100-200 nM, independent of mono- or co-culture status. Finally, ad-mixing CAFs (but not NFs) to H1437 cultures in collagen or mixed matrix provided resistance to the compound, with IC_50_s >500 nM (Table S2; S7B left panel). The latter finding is confirmed by a significant increase of EdU positive nuclei in GSK1059615 treated CAF co-cultures compared to mono, or NF co-cultures (Fig. S7B, right panel).

In conclusion, H1437 responses to GSK1059615 were affected in varying ways by culture formats, choice of matrices in embedded cultures, as well as by the ratio, and type of stromal cells in co-cultures.

### Image analysis of 3D cultures

***Image analysis of H1437 lung and LNCaP prostate cancer models***

As found in the size analysis for the breast cancer models, prostate and lung cancer floater cultures formed the largest spheroids. Other than for the LNCaP prostate models, the stromal cells produced a significant increase of lung floater spheroid sizes, confirming results from the growth curves presented above (Fig. S10; compare to Fig. S6/8). Matrigel- and collagen-embedded prostate cultures formed slightly larger spheroids than those embedded in the mixed matrix, but no difference was found for the lung. No effect of co-cultures was observed, as also found using growth curves (Fig. S10; compare to Fig. S6/8).

No analysis of EdU incorporation into lung and prostate spheroids was performed. For lung samples, image quality was not sufficient, whereas for the prostate samples the number of EdU-positive nuclei was too low (mean EdU positive cell ratio <0.005%).

### Analysis of tissue micro-arrays (TMA)

***TMAs of LNCaP prostate tumor models***

Collagen- and mixed-matrix embedded LNCaP cultures showed very low Ki67 counts with very high standard deviations (1.7 ±1.2% and 6.6 ±5.7% for mixed and collagen cultures, respectively), corroborating the above described fluorescence image analysis, which showed very low EdU incorporation into prostate tumor models. For spheroids, the numbers were slightly higher (17.5 ± 7.9%). Larger spheroids developed an apoptotic core, as demonstrated by cleaved caspase 3 staining (Fig. S11A).

The outgrowth of fibroblasts observed via fluorescence growth curves and images of SOC treated LNCaP/WPMY-1 co-cultures could be confirmed (Fig. S11B). In some of the LNCaP spheroids, cells expressed vimentin, even in LNCaP mono-cultures (Fig. S11C). This suggested a local induction of an epithelial-mesenchymal transition (EMT) and was observed in floaters as well as embedded cultures. Aggressive LNCaP sublines had been shown to express vimentin ^4^.

***TMAs of H1437 lung tumor models***

TMA analysis of lung 3D tumor models helped to address some issues that could not be resolved by fluorescence imaging.

H&E stained TMA sections from control and Docetaxel or GSK1059615 treated alginate-BR cultures revealed wide-spread necrotic areas in the treated spheroids (S12A). While the size of these spheroids remains unaltered, the fluorescence intensity will be expected to drop due to the loss of viable cells. This observation provides an explanation for the discrepancy between fluorescence-based growth curves, which showed an effect of the treatment (especially Docetaxel) and size analysis, which suggested no effect (Fig. S8C vs S12B).

As in the breast cancer floater model, fibroblasts were found in the core of lung floaters, indicating they formed inside-out spheroids (Fig. S13A). Similar islands of fibroblasts surrounded by tumor cells were also found in NSCLC patients (Fig. S13B).

### References

1 Veldscholte, J. *et al.* The androgen receptor in LNCaP cells contains a mutation in the ligand binding domain which affects steroid binding characteristics and response to antiandrogens. *The Journal of steroid biochemistry and molecular biology* **41**, 665-669 (1992).

2 Webber, M. M. *et al.* A human prostatic stromal myofibroblast cell line WPMY-1: a model for stromal-epithelial interactions in prostatic neoplasia. *Carcinogenesis* **20**, 1185-1192 (1999).

3 Lotti, F. *et al.* Chemotherapy activates cancer-associated fibroblasts to maintain colorectal cancer-initiating cells by IL-17A. *The Journal of experimental medicine* **210**, 2851-2872, doi:10.1084/jem.20131195 (2013).

4 Singh, S. *et al.* Overexpression of vimentin: role in the invasive phenotype in an androgen-independent model of prostate cancer. *Cancer research* **63**, 2306-2311 (2003).

5 Dolznig, H. *et al.* Characterization of cancer stroma markers: in silico analysis of an mRNA expression database for fibroblast activation protein and endosialin. *Cancer immunity* **5**, 10 (2005).

### Supplementary tables

**Table** S1: instruments

| **Culture** | | **Plate Reader** | | **Fluorescence Microscope** | | |
| --- | --- | --- | --- | --- | --- | --- |
| **Pathology** | **Platform** | **Instrument** | **Filters^1^** | **Instrument** | **Lenses (NA)** | **Filters** |
| Breast | 2D | BioTek (Synergy HT) | RFP: 530/25;  --; 590/35;  GFP:  485/20; --; 528/20 | Olympus (IX51) | 10x UPlanFL N (0.3) | BP 360-370, BP 470-495, BP 535-555HQ |
|  | 3D BR | Tecan (Infinite M200) | 488; --; 509 nm | Andor (Revolution WD Spinning Disk) | 10x Plan Fluor (0.3)  20x Plan Fluor (0.75) | 405, OPSL CW 488, DPSS 561 and DPSS 640 |
|  | 3D floaters | Tecan  (Infinite M200) | 485; --; 535 nm | Zeiss  (LSM700) | 10x EC PLAN NEOFLUAR M27 (0.3) | 669; 555; 405; 488; BF |
|  | 3D matrix |  |  |  |  |  |
| Prostate | 2D | Molecular Devices  (SpectraMax M2e) | RFP: 540; 570; 587;  GFP: 488; 515; 525 nm | Life Technologies (EVOS FL Imaging System) | 10x Plan Achromat (0.25) | BP 470/22-510/42; BP 531/40-593/40; BF |
|  | 3D floaters |  |  | Zeiss  (LSM510 META Multiphoton) | 10x Plan Apochromat (0.45) | BP 500-530 IR; BP 390-465 IR (two photon); BP 565-615 IR; BP 650-710 IR |
|  | 3D  matrix |  |  | Yokogawa  (CV 7000 Spinning Disk) | 10x UPLSAPO (0.4) | BP 600/37; BP 522/35; BP 447/45; BP 676/29 |
| Lung | 2D | Tecan  (Infinite M200)  PE-Wallach  (EnVision 2100) | 485; 531 nm | Nikon  (eclipse Ti-E)  ZEISS  (Axiovert 200M) | 10x A-Plan (0.25) | 489; 546; BF |
|  | 3D BR | Tecan (Infinite M200) |  | Andor  (Revolution WD  Spinning Disk) | 10x Plan Fluor (0.3)  20x Plan Fluor (0.75) | 405, OPSL CW 488, DPSS 561 and DPSS 640 |
|  | 3D  floaters | PE-Wallach  (EnVision 2100) | 485; 531 nm | ZEISS  (Axiovert 200M) | 10x A-Plan (0.25) | 489; 546; BF |
|  | 3D  matrix | PE-Wallach  (EnVision 2100) | 485; 531 nm | Zeiss  (Axiovert 200M) | 10x A-Plan (0.25) | 489; 546; BF |

Table S2. Treatment schedules and IC50 values:

| **MODEL** | | **TREATMENT** | | | | **IC_50_** |
| --- | --- | --- | --- | --- | --- | --- |
| **Pathology** | **Platform** | **Drug** | **Dose range** | **Start**  **(day)** | **Time (days)** | **Mono, Co**  **(nM)** |
| Breast | 2D | Fulvestrant | 0.3 pM- 1 µM | 2 | 9 | M: 0.2  C: 0.2 |
|  |  | Docetaxel | 0.3 pM-1 µM |  |  | M: 0.4  C: 0.4 |
|  | Floaters | Fulvestrant | 0.1 pM- 1 µM | 3 | 6 | M: 0.4  C: 0.6 |
|  |  | Docetaxel | 0.1 pM- 1 µM |  |  | M: 7.6  C: 13 |
|  | BR | Fulvestrant | 0.1 pM- 1 µM | 6 | 15 | M: 0.4  C: 0.4 |
|  |  | Docetaxel | 0.1 pM- 1 µM |  |  | M: 7.3  C: 7.3 |
|  | Matrix | Fulvestrant | 1 pM- 1 µM | 7 | 9 | Matrigel  M: 2.5  C: 2.8 |
|  |  |  |  |  |  | Collagen  M: 0.9  C: 1 |
|  |  |  |  |  |  | Mix  M: 1.1  C: 1.1 |
|  |  | Docetaxel | 1 pM- 1 µM |  |  | Matrigel  M: 3.2  C: 4.8 |
|  |  |  |  |  |  | Collagen  M: 0.9  C: 0.8 |
|  |  |  |  |  |  | Mix  M: 1.3  C: 1.7 |
| Prostate | 2D | MDV-3100 | 0.01 – 10 µM | 2 | 11 | M: 73  CC: 62  CW: 41 |
|  |  | Docetaxel | 0.3 – 300 nM |  |  | M: 0.8  CC: 0.86  CW: 0.67 |
|  | Floaters | MDV-3100 | 0.01 – 10 µM | 6 | 15 | M: 166  CC: 109  CW: 47 |
|  |  | Docetaxel | 0.3 – 300 nM |  |  | M: 14.3  CC: 13.9  CW: 9.6 |
|  | Matrix | MDV-3100 | 0.01 – 10 µM | 6 | 13-16 | Matrigel  M: 195  CC: 101  CW: 244 |
|  |  |  |  |  |  | Collagen  M: 30  CC: 47  CW: 27 |
|  |  |  |  |  |  | Mix  M: 79  CC: 17  CW: 126 |
|  |  | Docetaxel | 0.3 – 300 nM |  |  | Matrigel  M: 3.5  CC: 1.2  CW: 1.9 |
|  |  |  |  |  |  | Collagen  M: 8.2  CC: 3.8  CW: 1.0 |
|  |  |  |  |  |  | Mix  M: 1.7  CC: 5.8  CW: 2.1 |
| Lung | 2D | GSK1059615 | 7.8 nM – 2 µM |  |  | M: 91.5  CC: 18  CN: 31.8 |
|  |  | Docetaxel | 7.8 nM – 2 µM |  |  | M: 13.2  CC: 9.8  CN: 7.8 |
|  | Floaters | GSK1059615 | 3 nM- 2 µM | 7 | 13 | M: 10.5  CC: 37.5  CN: 58.4 |
|  |  | Docetaxel | 8 nM- 1 µM |  |  | M: 27.4  CC: 10.8  CN: 15.7 |
|  | BR | GSK1059615 | 0.1 nM- 10 µM | 6 | 10 | M: 900  CC: 280  CN: 500 |
|  |  | Docetaxel | 0.001nM- 1 µM |  |  | M: 390  CC: 99  CN: 56 |
|  | Matrix | GSK1059615 | 7.8 nM – 2 µM | 7 | 10 | Matrigel  M: 139.1  CC: 124.5  CN: 176.8 |
|  |  |  |  |  |  | Collagen  M: 24.9  CC: 785.5  CN: 66.3 |
|  |  |  |  |  |  | Mix  M: 31.7  CC: 523.6  CN: 30.4 |
|  |  | Docetaxel | 7.8 nM – 2 µM |  |  | Matrigel  M: 13.2  CC: 117.9  CN: 11.1 |
|  |  |  |  |  |  | Collagen  M: 9.8  CC: 24.2  CN: 93.7 |
|  |  |  |  |  |  | Mix  M: 14.8  CC: 24.6  CN: 12.2 |


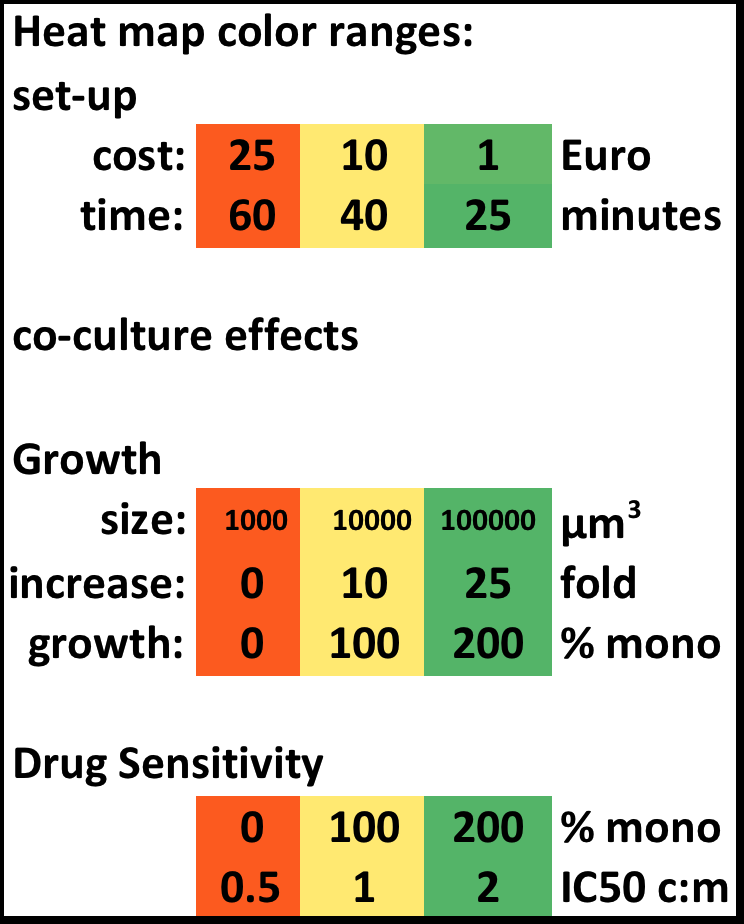
**Table S3: overview on model cost and effect on tumor cell growth and resistance**


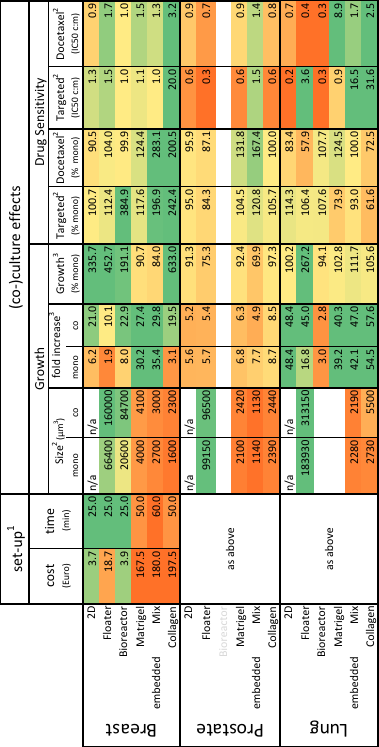


1 - for one 96 well plate (or equivalent); 2 - at endpoint; 3 - from day 0 to endpoint;

### Supplementary Figures

#### Figure index

Figure S1. High magnification images of MCF7-NDF floaters.

Figure S2. Protective effect of HDF on MCF7 SOC treatment.

Figure S3. LNCaP prostate tumor model characterization using fluorescence-based growth curves.

Figure S4. H1437 lung model characterisation using fluorescence-based growth curves.

Figure S5. Effect of stroma:tumour ratio, and fibroblast type, on growth of 2D and alginate-BR lung H1437 models.

Figure S6. Response of LNCaP prostate model platforms to SOC treatment.

Figure S7. Prostate/lung pathology-specific observations.

Figure S8. Fluorescence-based growth curves of the NCI-H1437 lung cancer models treated with GSK1059615 or Docetaxel.

Figure S9. Effect of stroma:tumor ratio, on response of H1437 lung 2D cultures to SOC treatment.

Figure S10. in situ image analysis & quantification (prostate & lung)

Figure S11. IHC staining of Ki67, cleaved caspase 3, AR, E-cadherin, and vimentin in TMA sections from LNCaP 3D cultures

Figure S12. Image-based size analysis compared to HE stains of FFPE material.

Figure S13. Lung cancer stromal cells in floaters and patient samples.

Figure S14. Type and ratio of fibroblasts in MCF7 models

#### Figure S1


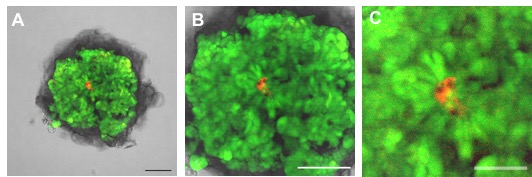


Figure S1. *High magnification images of MCF7-HDF floaters.*

MCF7 tumor cells were co-cultured with HDFs in the floater model, and live-cell imaging was performed on day 9, as in figure 2B/H. In A-C, increasing magnifications are shown. GFP-labeled tumor cells in green, RFP-labeled HDF stromal fibroblasts in red. Scale bars: A, B: 100 µm; C: 50 µm

#### Figure S2


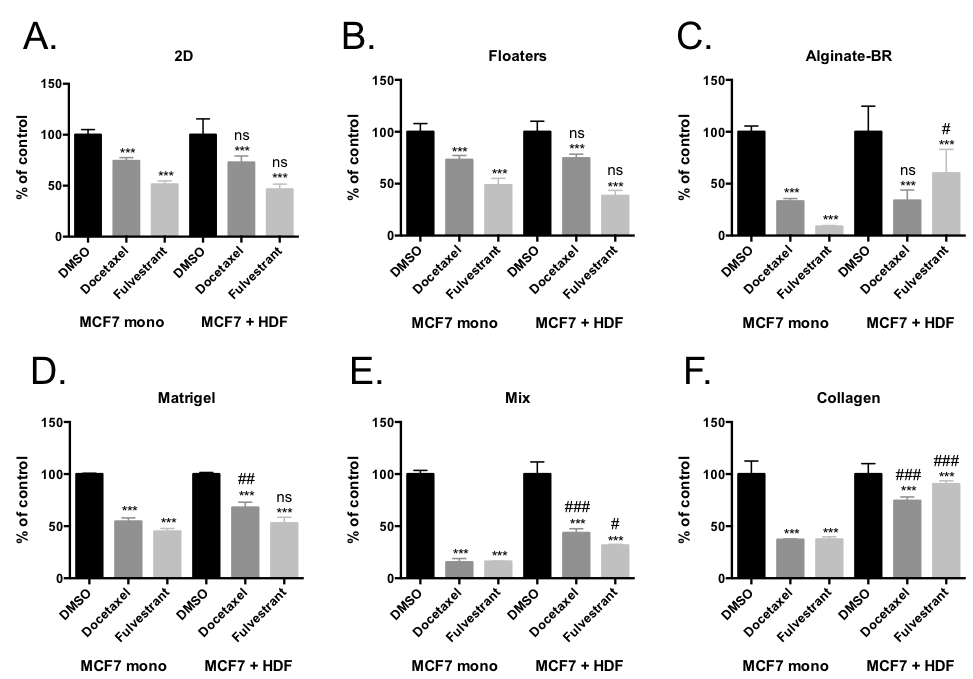


Figure S2. *Protective effect of HDF on MCF7 SOC treatment*.

Models were set up, and growth of MCF7 tumor cells was measured as described in M&M. Mono- and co-cultures were treated with Fulvestrant (F) or Docetaxel (D) at concentrations corresponding to IC_80_ or IC_50_ of the compounds on the mono-cultures, respectively. Bar graphs show remaining fluorescence at endpoint, relative to DMSO treated control cultures. Significances are indicated for comparison to DMSO treated controls (*) or monocultures (#) (ns: not significant; *p < 0.05; **p < 0.01; ***p < 0.001) . A. 2D culture (D: 0.3 nM; F: 0.3 nM); B. Floaters (D: 10 nM; F: 1 nM); C. Alginate-BR (D: 7.3 nM; F: 1 nM); D. Matrigel (D: 3 nM; F: 10 nM); E. Mix (D: 3 nM; F: 3 nM); F. Collagen (D: 1 nM; F: 0.3 nM)

#### Figure S3


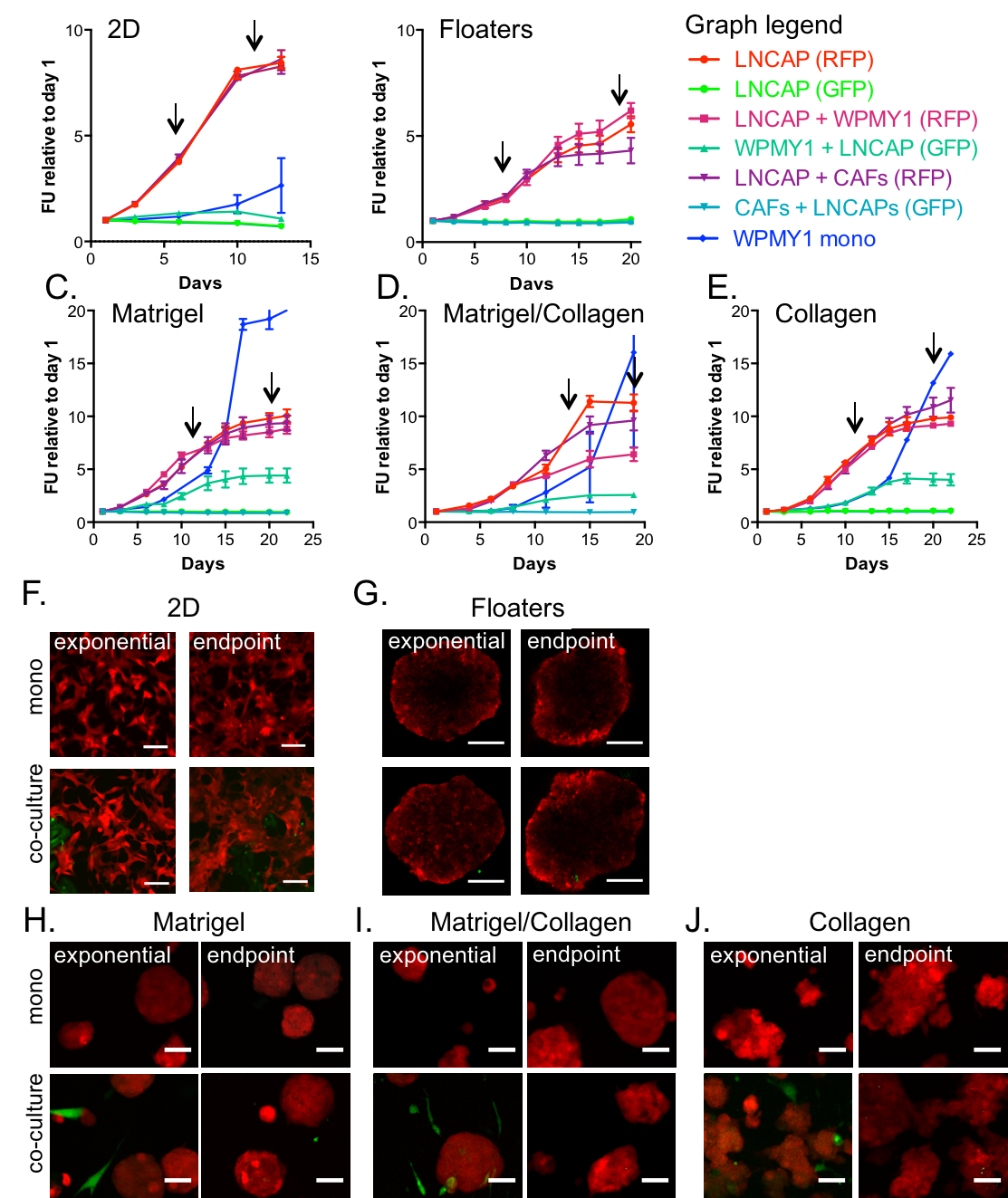


Figure S3. *LNCaP prostate tumor model characterization using fluorescence-based growth curves*.

Models were set up, and growth of LNCaP tumor cells was measured via RFP fluorescence, of WPMY1 and CAF stromal fibroblasts using GFP fluorescence, as described in M&M. Only WPMY1 stromal mono-cultures proliferated in this set-up. The graphs in the upper panels show growth curves of LNCaP in mono-cultures as a red line, measured in the green channel, as a negative control, as a light green line. In WPMY1 co-cultures, the LNCaP signal is in pink, WPMY1s are in dark green, and in CAF co-cultures LNCaPs are violet, and CAFs blue-green. WPMY1 mono-cultures are shown in blue. Arrows indicate time-points when pictures were taken. (A), 2D, (B), floaters, (C) Matrigel, (D) Matrigel/collagen, (E) collagen models. In F-J, fluorescence images corresponding to A-E are shown. RFP labeled tumor cells in red, GFP-labeled stromal fibroblasts in green. Scale bars: 2D: 100 µm, floaters: 200 µm; embedded: 50 µm.

Figure S4

***
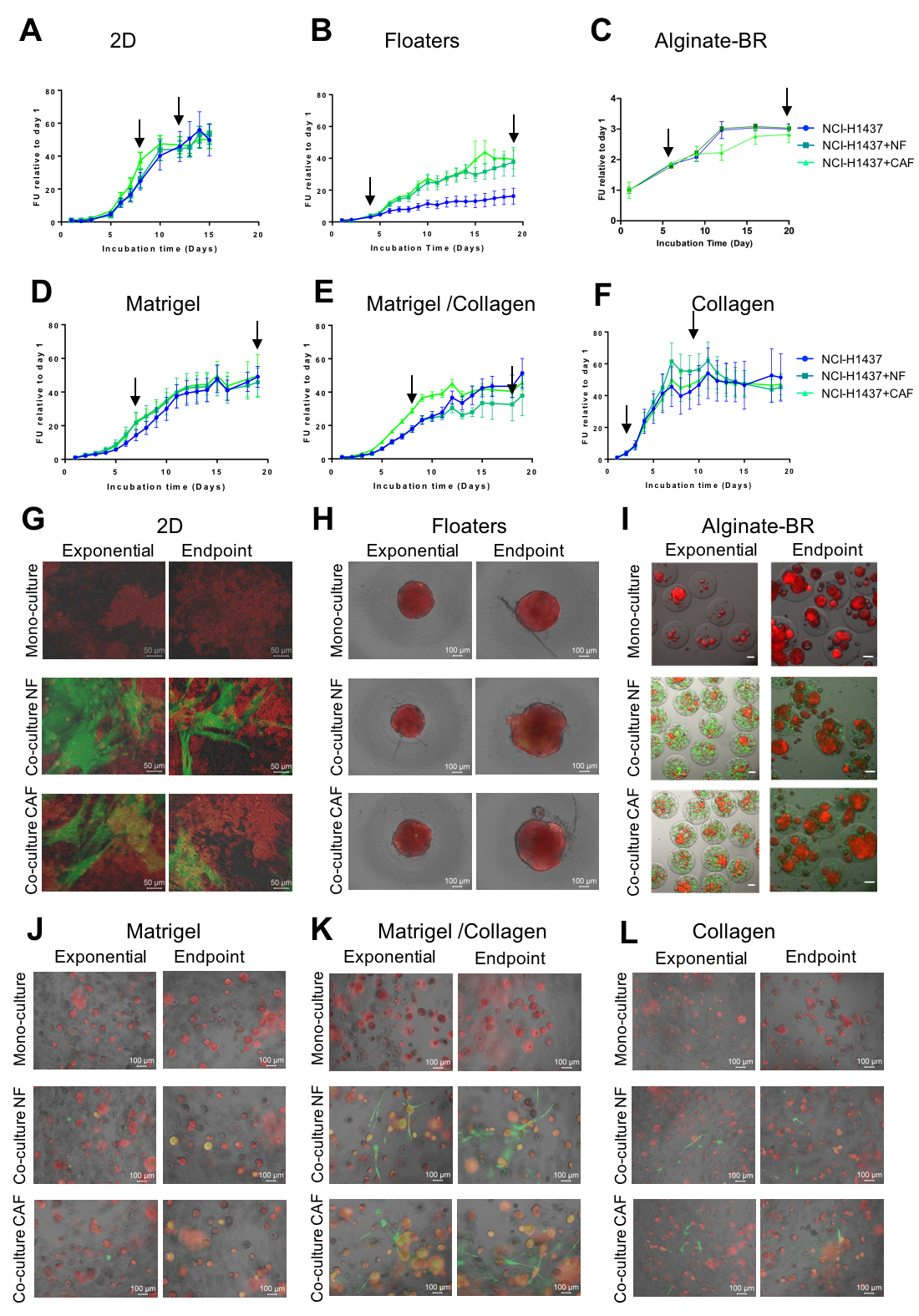
***

Figure S4. *H1437 lung model characterisation using fluorescence-based growth curves*.

Models were set up, and growth of NCI-H1437 tumor cells was measured via RFP fluorescence as described in M&M. Stromal cell growth could not be detected. The graphs in the upper panels show growth curves of the mono- (blue), NF co-cultures (dark green), and CAF co-cultures (light green), grown in (A), 2D, (B), floaters, (C), alginate-BR, (D) Matrigel, (E) Matrigel/collagen, (F) collagen. Arrows indicate time-points when pictures were taken. In G-L, fluorescence images corresponding to A-F are shown. RFP labeled tumor cells in red, GFP-labeled HDF stromal fibroblasts in green. Scale bars: 2D, alginate-BR: 50 µm; floaters, embedded: 100 µm.

#### Figure S5


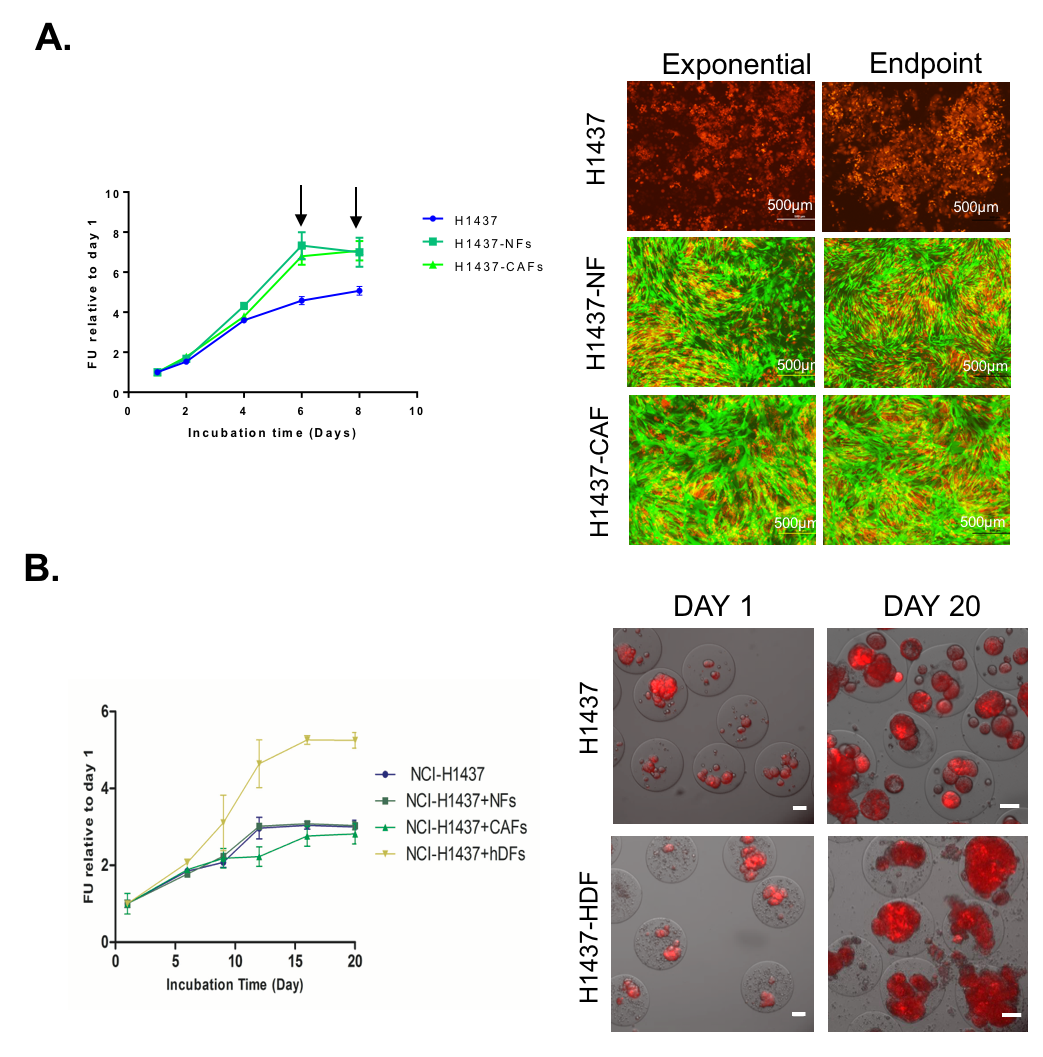


Figure S5*. Effect of stroma:tumor ratio, and fibroblast type, on growth of 2D and alginate-BR lung H1437 models.*

Models were set up, and growth of H1437 tumor cells was measured as described in M&M. A. 2D cultures with a 1:1 ratio of tumor: NFs and CAFs. Left panel, graphs for mono-culture (blue) and co-cultures (dark green – NF; light green CAF). The right panel shows corresponding fluorescence images. B. Alginate-BR H1437 cultures grown as mono-, or NF, CAF, or HDF co-cultures, at a ratio of 1:1. Left panel, graphs for mono-culture (blue) and co-cultures (dark green – NF; light green CAF; brown - HDF). The right panel shows fluorescence images of HDF co-cultures.

#### Figure S6


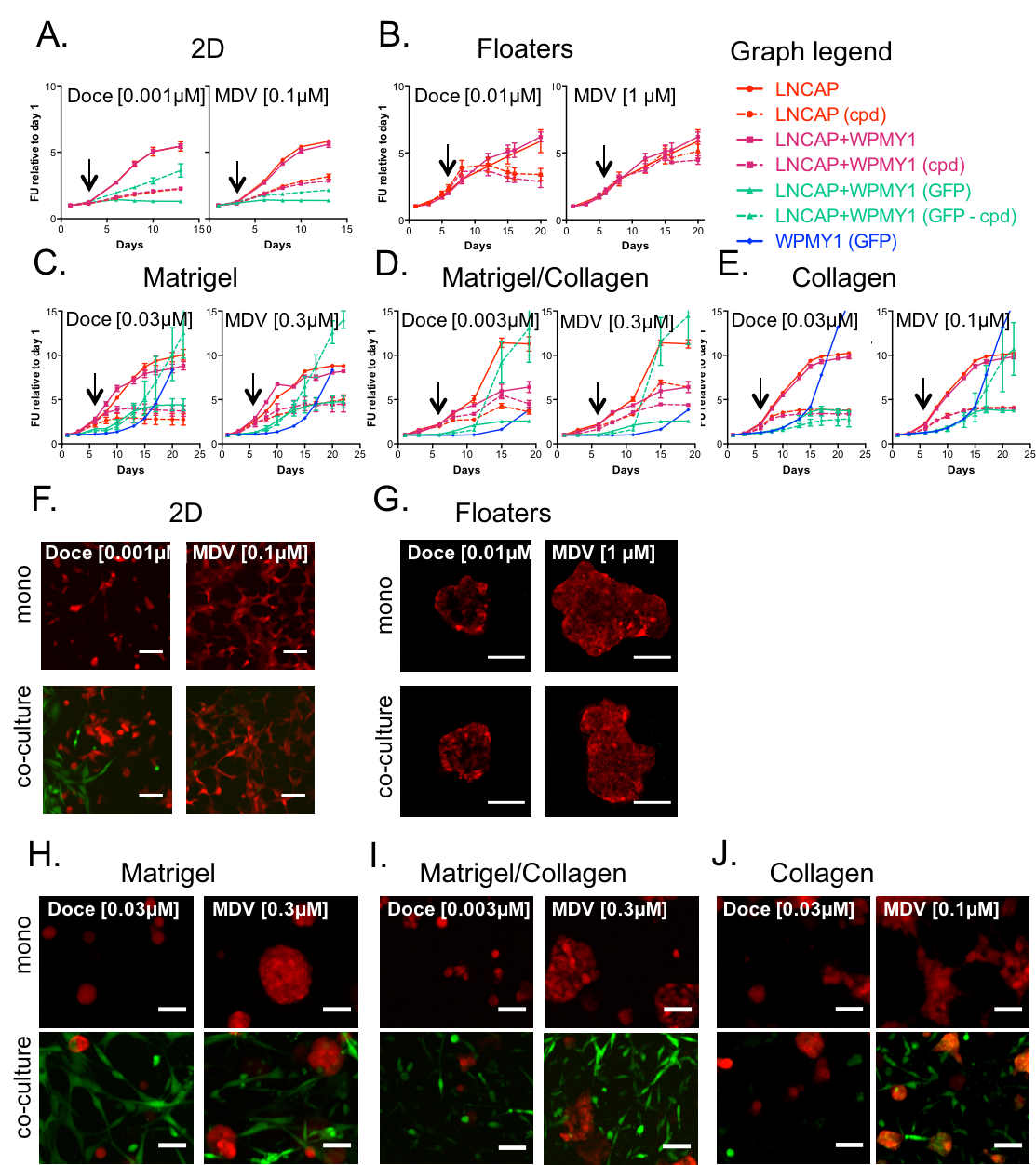


Figure S6. *Response of LNCaP prostate model platforms to SOC treatment*.

Models were set up, and growth of LNCaP tumor cells and CAF/WPMY1 fibroblasts was measured as described in M&M. Mono- and co-cultures were treated with MDV-3100 or Docetaxel at the concentrations in the fluorescence images, corresponding to IC_80_ and IC_50_ values on mono-cultures. Only WPMY1 co-culture results are included - CAFs behaved similarly, except for no increase of the GFP signal was observed over time. Untreated (DMSO) controls are straight, treated broken lines. The signal from the RFP labeled LNCaP in mono-cultures is shown as a red line, in WPMY1 co-cultures in pink (RFP), with the WPMY1s in dark green (GFP). WPMY1 mono-cultures are shown in blue (GFP). Depicted are, in (A), 2D, (B), floaters, (C), Matrigel, (D) Matrigel/collagen, (E) collagen cultures. Arrows indicate start of treatment. In F-J, fluorescence images corresponding to A-E are shown. RFP labeled LNCaP tumor cells in red, GFP-labeled WPMY-1 fibroblasts in green. Scale bars: 2D: 100 µm, floaters: 200 µm; embedded: 50 µm.

#### Figure S7


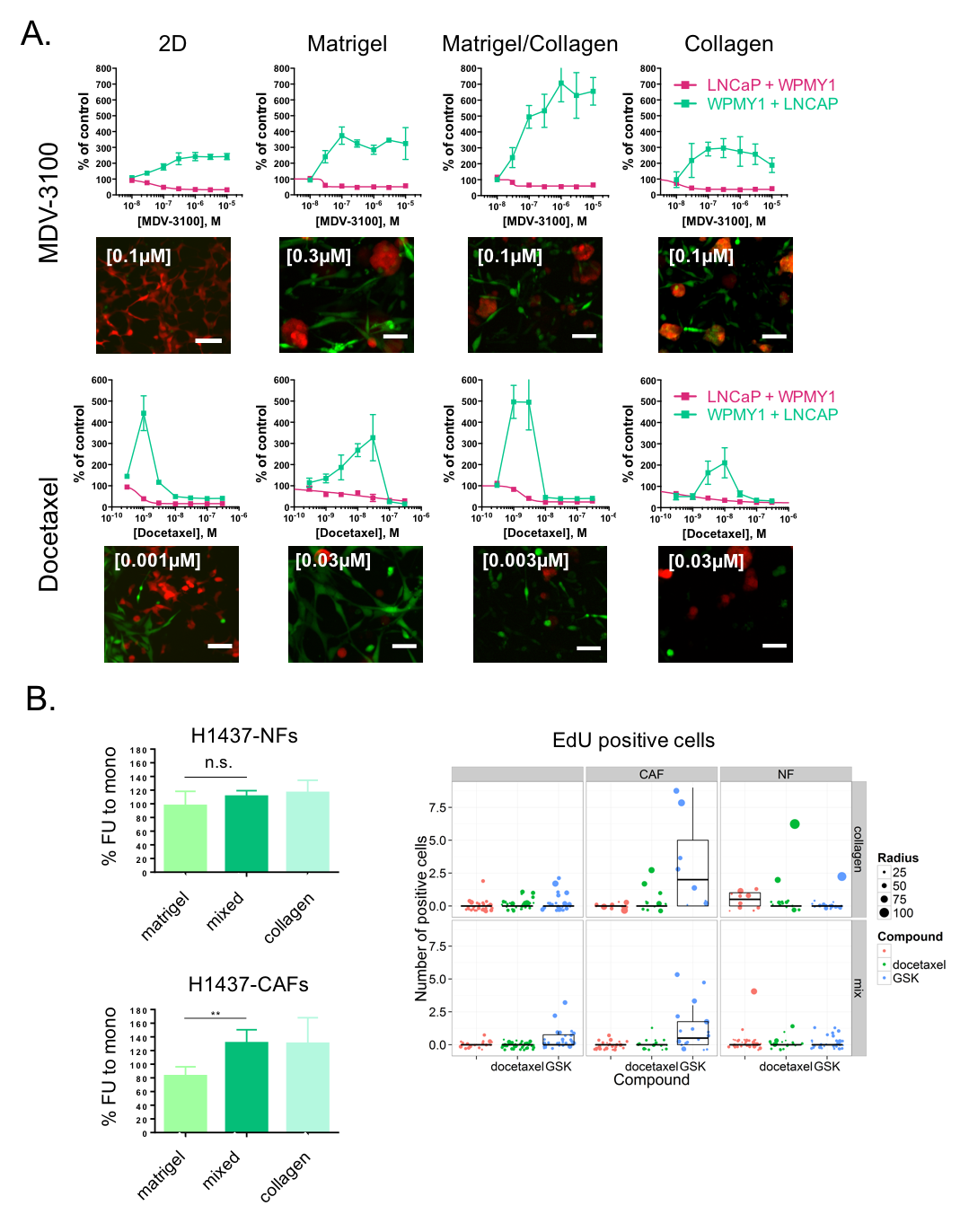


Figure S7. *Prostate/lung pathology-specific observations*.

A. Outgrowth of fibroblasts in treated 2D and 3D embedded prostate co-cultures.

Models were set up, and growth of LNCaP tumor cells and WPMY-1 fibroblasts was measured as described in M&M. Cultures were treated with serial dilutions of 10 µM-10 nM MDV-3100 (upper panel) or 100 nM-0.1 nM Docetaxel (lower panel). Dose response curves were generated for LNCaP (purple)/WPMY1 (green) co-cultures. Note the increase of WPMY1 fluorescence with increasing drug concentrations (see text for more explanation). Below the graphs, corresponding representative fluorescence images are shown. RFP labeled tumor cells in red, GFP-labeled WPMY1 fibroblasts in green. Scale bars: 2D: 100 µm, embedded: 50 µm.

B. Protective effect of CAFs on GSK1059615 treated H1437 mixed and collagen embedded cultures.

On the left, bar graphs comparing the fluorescence units (FU) of H1437 NF or CAF co-cultures treated with 32 nM GSK1059615 (corresponding to IC_80_) with mono-cultures. Note the matrix-dependent protective effect of the CAFs (but not NFs). On the right, similar cultures were stained for proliferating cells with EdU. CAFs significantly increased the number of proliferating cells in GSK1059615 treated H1437 co-cultures (blue), but not Docetaxel treated (green) co-cultures.

#### Figure S8


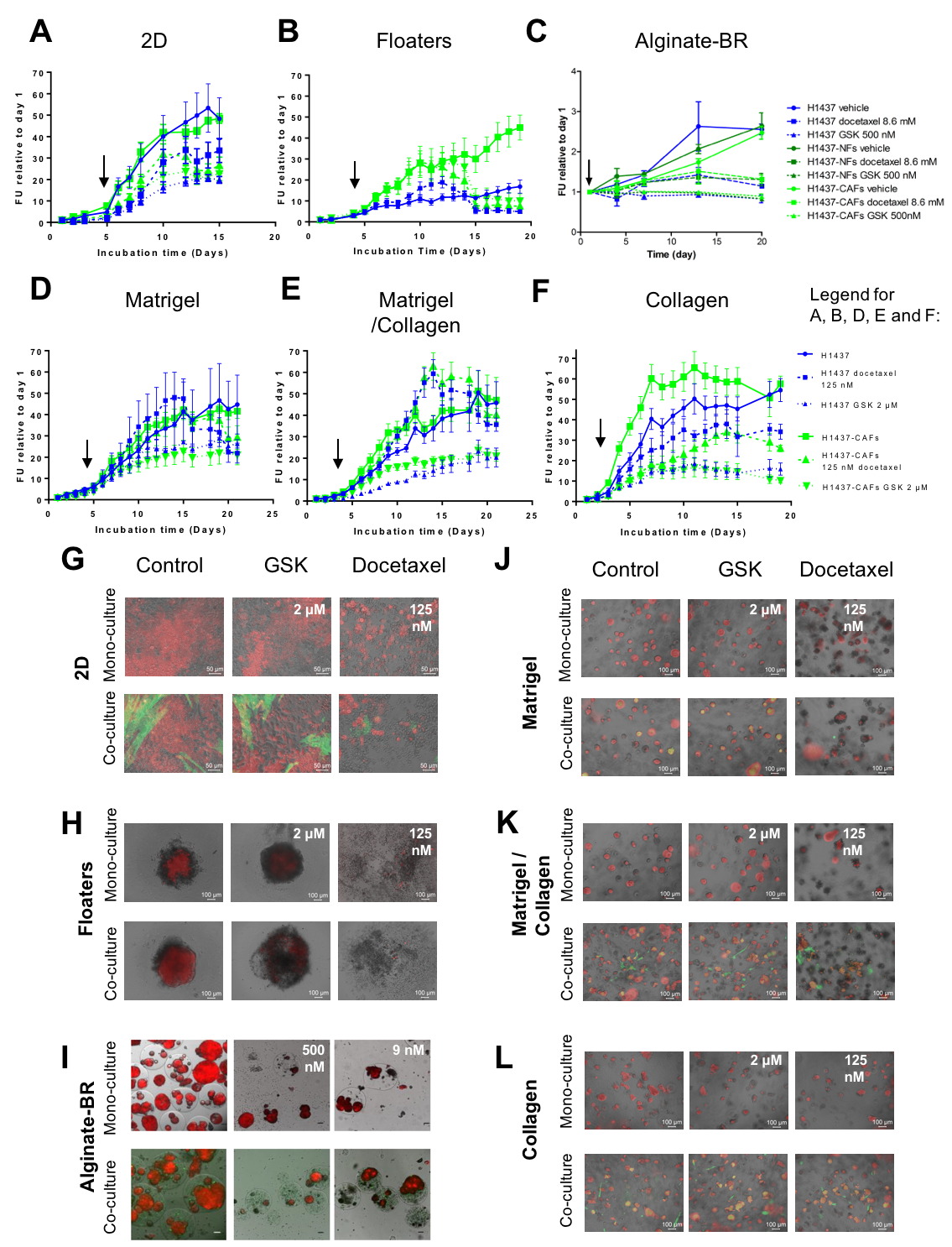


Figure S8. *Fluorescence-based growth curves of the NCI-H1437 lung cancer models treated with GSK1059615 or Docetaxel*.

Models were set up, and growth of NCI-H1437 tumor cells was measured as described in M&M. Stromal cell growth could not be detected. Mono- and co-cultures were treated with GSK1059615 or Docetaxel at the concentrations indicated in the fluorescence images (G-L), corresponding to IC_80_ and IC_50_ values on mono-cultures. Untreated cultures (DMSO control) are indicated as straight, GSK1059615 with a dotted, and Docetaxel with a broken line. Shown are the graphs from H1437 as mono-cultures (blue), as NF co-cultures (dark green), and as CAF co-cultures (light green) grown in (A), 2D, (B), floaters, (C), alginate-BR, (D) Matrigel, (E) Matrigel/collagen, (F) collagen. Arrows indicate start of treatment. In G-L, fluorescence images corresponding to A-F are shown. RFP labeled tumor cells in red, fibroblasts in green. Scale bars: 2D: 100 µm, floaters: 200 µm; embedded: 50 µm.

#### Figure S9


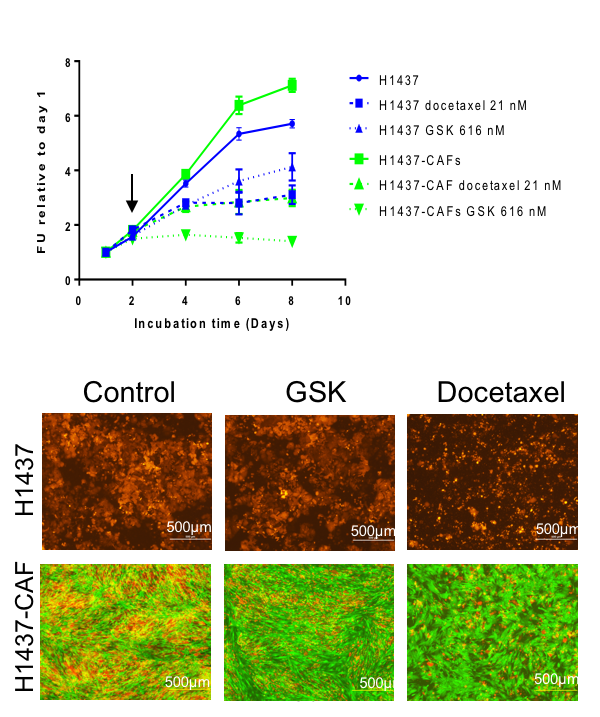


Figure S9*. Effect of stroma:tumor ratio, on response of H1437 lung 2D cultures to SOC treatment*.

Models were set up, and growth of H1437 tumor cells was measured as described in M&M. H1437 mono-cultures (blue), CAF co-cultures (light green) at a ratio of 1:1 were grown in 2D and treated with with 21 nM docetaxel or 616 nM GSK1059615, corresponding to IC_80_ or IC_50_ concentrations of the compounds on the mono-cultures, respectively. Untreated (DMSO control) are shown as straight lines, treated cultures as dotted lines. Note that Docetaxel treated mono-and CAF-related lines are overlapping. The lower panel shows corresponding fluorescence images.

#### Figure S10


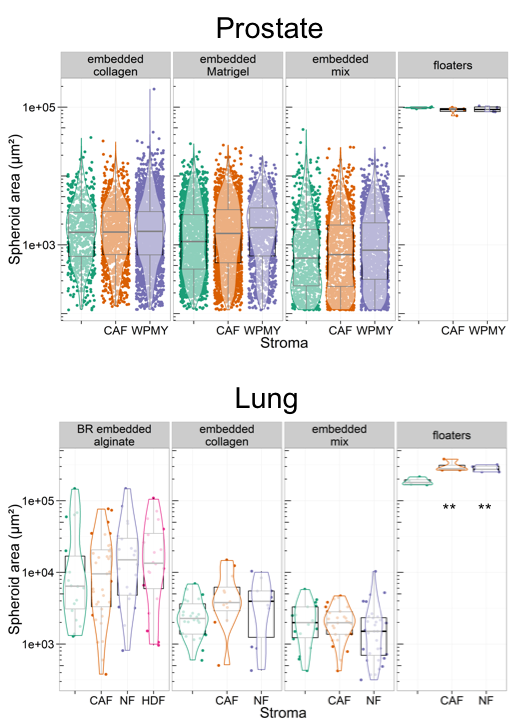


Figure S10**.** in situ *image analysis & quantification (prostate & lung)*

Scatter plots comparing LNCaP (prostate) and H1437 (lung) 3D models by spheroid sizes. Mono- (green) and co-culture (orange (CAFs) and purple (WPMYs or NFs (lung)) spheroid areas derived from MIP analyses are displayed on a logarithmic scale.

*-symbols compare co- with mono-cultures.

#### Figure S11


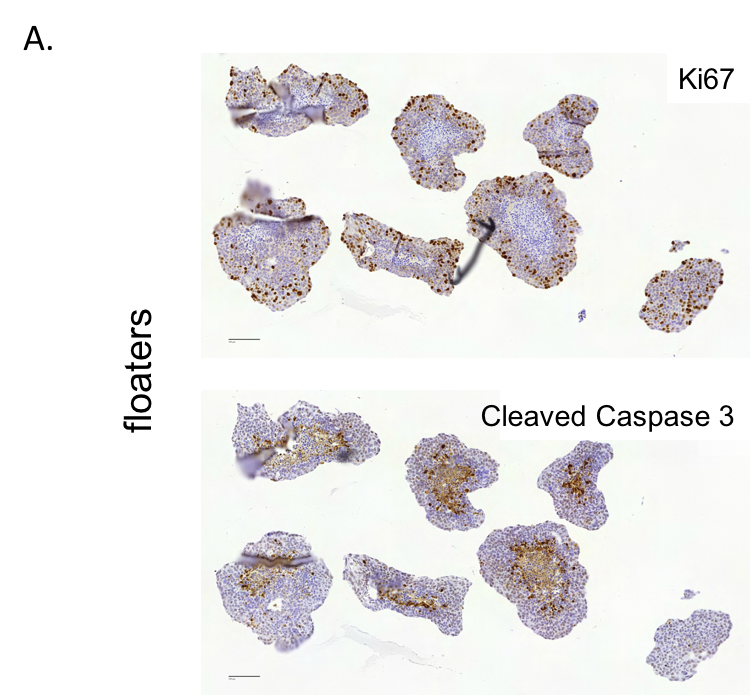


Figure S11. *IHC staining of Ki67, cleaved caspase 3, AR, E-cadherin, and vimentin in TMA sections from LNCaP 3D cultures*

A. Sections from LNCaP/CAF co-culture floater FFPE samples were stained for Ki67 and cleaved caspase 3, and counterstained with hematoxylin. Note Ki67 positive cells around the rim and necrotic cores in larger spheroids.

B. Sections with control, Docetaxel or MDV-3100 treated collagen or mixed matrix LNCaP/WPMY1 co-culture FFPE samples were stained for AR, E-Cadherin, and Vimentin, and counterstained with hematoxylin. Upper panels, collagen embedded. Docetaxel: 0.03 µM; MDV: 0.1 µM B. Lower panels: mixed matrix embedded. Docetaxel: 0.003 µm; MDV: 0.1 µM. Note the increase of Vimentin-positive single cells in drug-treated co-cultures.

C. Sections of LNCaP mono- and WPMY1 co-culture FFPE samples were stained for E-Cadherin and Vimentin, and counterstained with hematoxylin. Upper panel: mixed matrix embedded; lower panel: floater cultures. Note Vimentin positive LNCaP cells.

Scale bars: 100 µm.


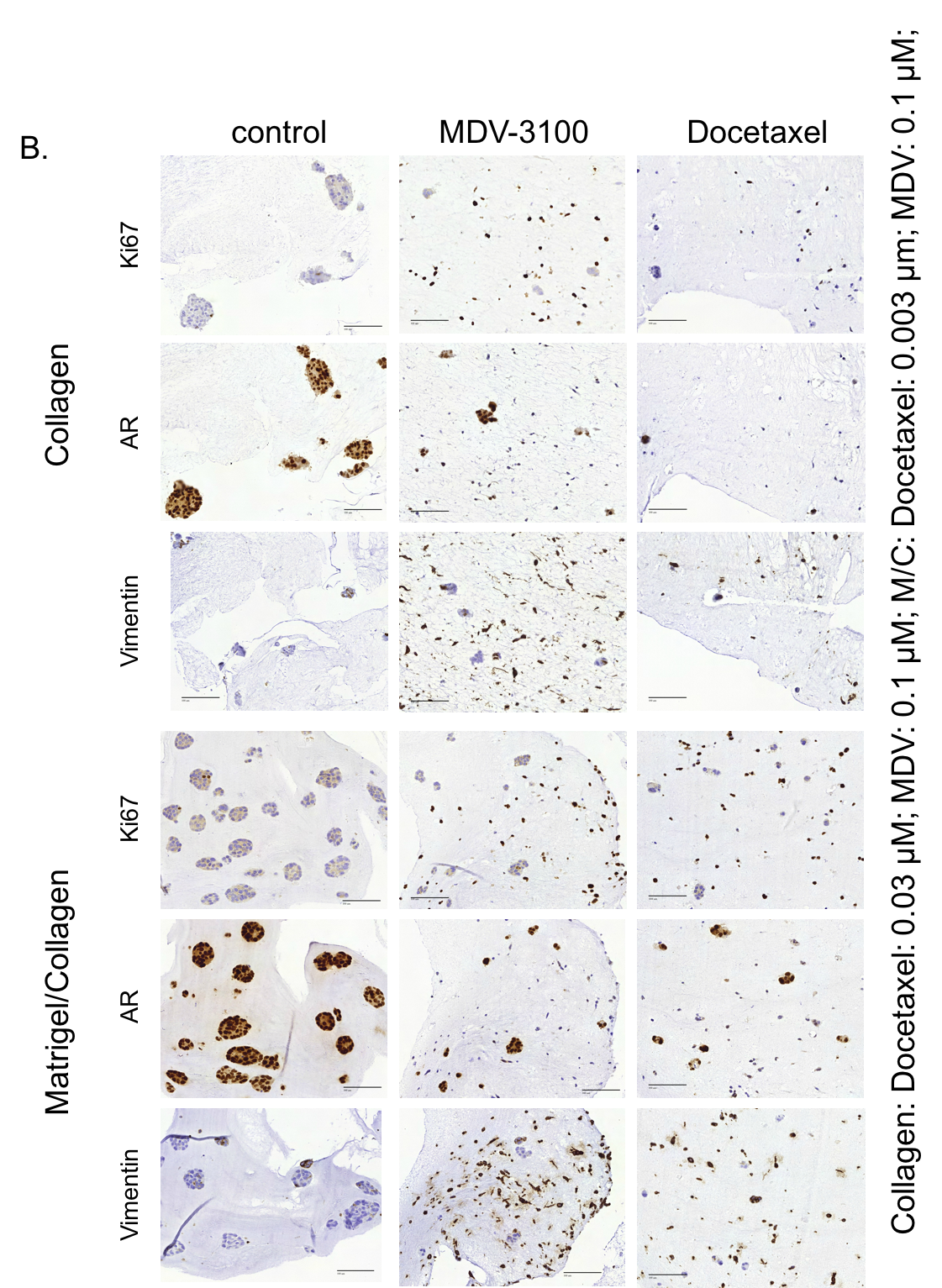


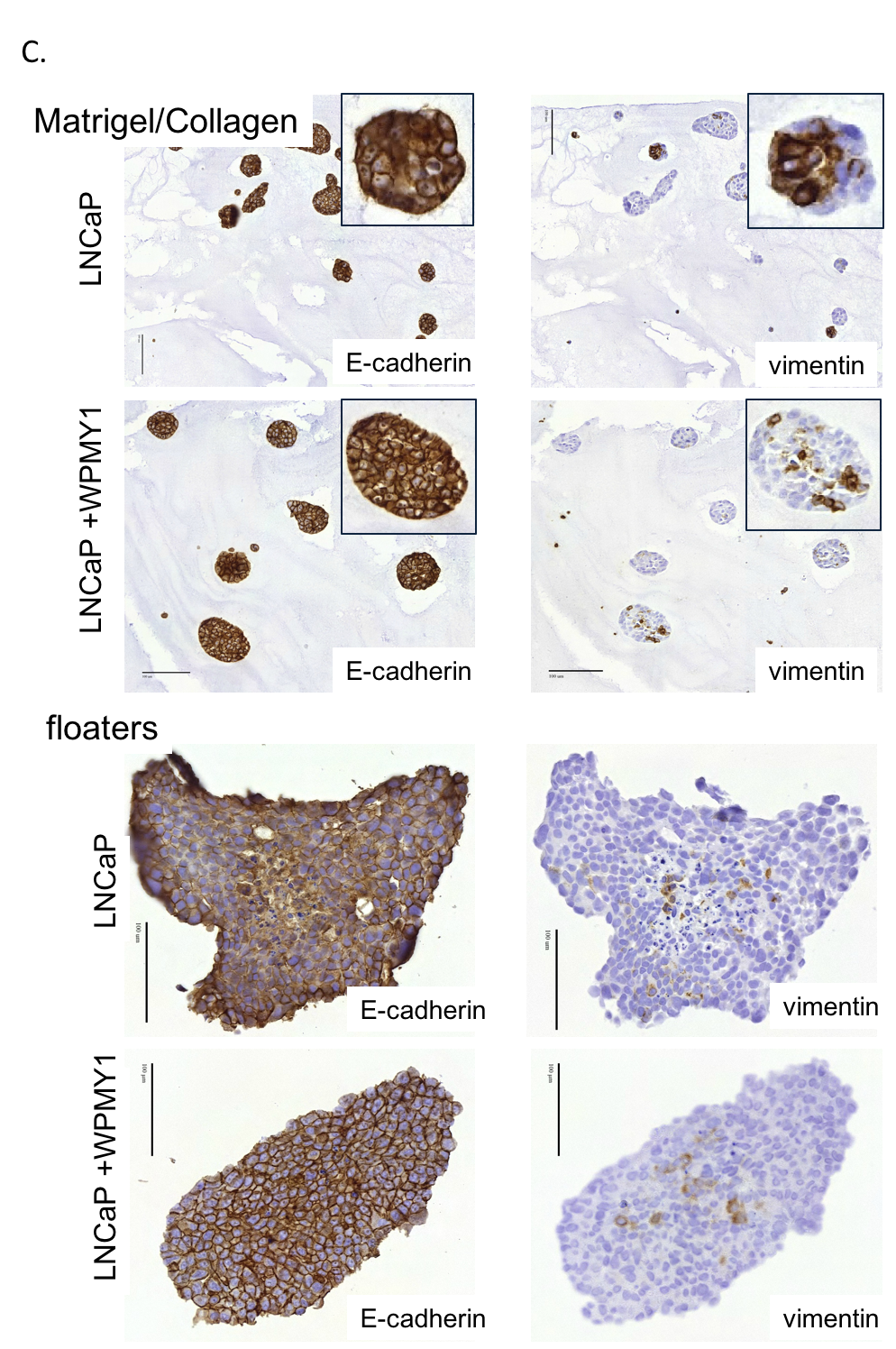


#### Figure S12


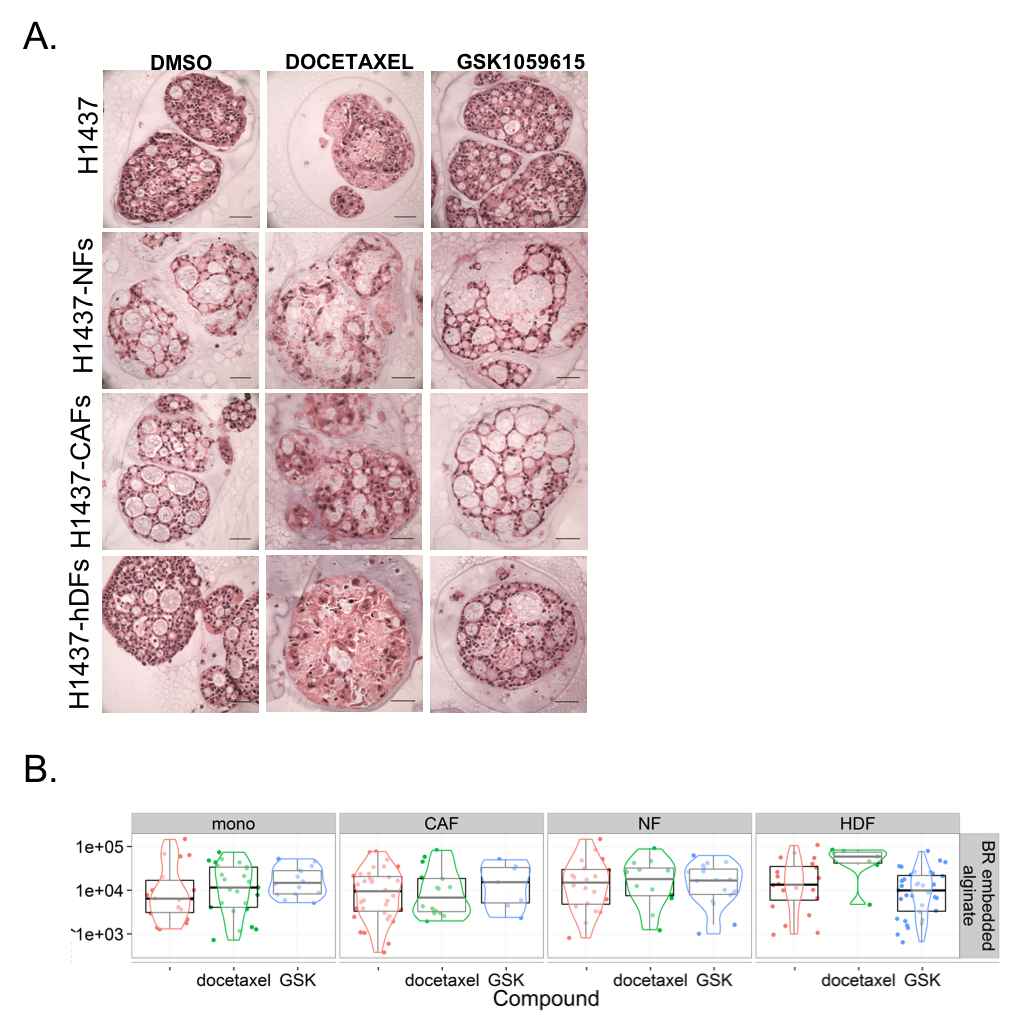


Figure S12. *Image-based size analysis compared to HE stains of FFPE material.*

A. HE stained FFPE alginate-BR cultures. Note the different morphology of the spheroids induced by the fibroblasts in the co-cultures, and the necrotic areas in Docetaxel treated cultures in the absence of an apparent shrinking of the spheroids.

B. Size analysis of images from lung alginate-BR mono- and co-cultures, as in figure S9. Note the apparent size increase of the Docetaxel treated HDF-co-cultures.

#### Figure S13


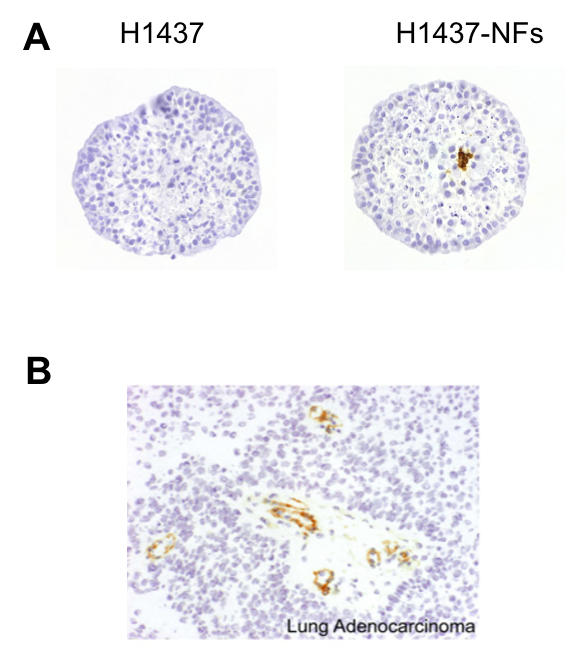


Figure S13. *Lung cancer stromal cells in floaters and patient samples.*

A. Vimentin staining of unperturbed monoculture (H1437) and co-culture (H1437-NFs) floaters revealed positive cells for co-culture TMAs of H1437-NFs at day 9.

B. Immunohistochemistry with mAb FB5 showed patterns of endosialin expression in lung adenocarcinoma in patients (from Dolznig and colleagues ^5^).

#### Figure S14


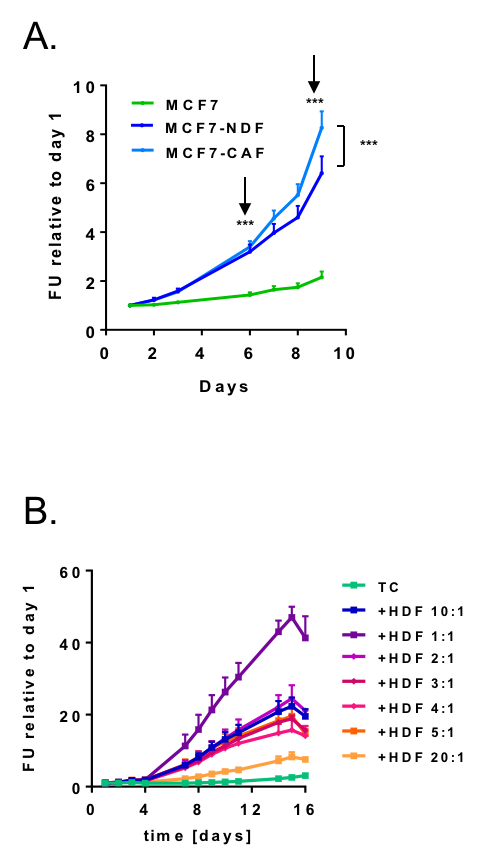


Figure S14. *Type and ratio of fibroblasts in MCF7 models*

*A. Comparison of the effect of HDFs and primary breast cancer associated fibroblasts on the MCF7 floater co-culture model*.

MCF7 tumor cells were co-cultured with breast cancer-associated fibroblasts (CAFs) or human dermal fibroblasts (HDF) in the 3D floater assay. Growth of the floaters was measured using GFP fluorescence as described in M&M. Note similar growth kinetics in both co-culture models until day 9, after which breast CAFs show a slight growth advantage (p<0.001).

*B. Titration of MCF7 tumor cell: HDF stromal cell ratios in a collagen embedded model*. MCF7 and HDF cells were embedded in collagen at ratios of 1:1- 20:1 (tumour:stroma), as sixplicates. Growth of the tumour cells was measured via GFP fluorescence intensity. Stromal cell growth could not be detected.

Left panel: Growth curve. Note the ratio-dependent effect of the stromal cells on the tumour cells.

Right panel: Collagen contraction by HDF stromal cells. At 1:1 and 2:1 tumor:stroma ratios, a marked collagen contraction was detected after 16 days in culture.
